# A systematic literature review of the global seroprevalence of cytomegalovirus: possible implications for treatment, screening, and vaccine development

**DOI:** 10.1186/s12889-022-13971-7

**Published:** 2022-09-01

**Authors:** Karen Fowler, Jacek Mucha, Monika Neumann, Witold Lewandowski, Magdalena Kaczanowska, Maciej Grys, Elvira Schmidt, Andrew Natenshon, Carla Talarico, Philip O. Buck, John Diaz-Decaro

**Affiliations:** 1 University of Alabama at Birmingham, Birmingham, AL USA; 2 Certara, Inc., Krakow, Poland; 3 Certara, Inc., Lörrach, Germany; 4 Moderna, Inc., 200 Technology Square, Cambridge, MA 02139 USA

**Keywords:** Cytomegalovirus, Congenital cytomegalovirus, CMV, Epidemiology, Prevalence, Seroprevalence, Shedding

## Abstract

**Background:**

Cytomegalovirus (CMV) is a common pathogen that affects individuals of all ages and establishes lifelong latency. Although CMV is typically asymptomatic in healthy individuals, infection during pregnancy or in immunocompromised individuals can cause severe disease. Currently, treatments are limited, with no prophylactic vaccine available. Knowledge of the current epidemiologic burden of CMV is necessary to understand the need for treatment and prevention. A systematic literature review (SLR) was conducted to describe the most recent epidemiologic burden of CMV globally.

**Methods:**

Medline, Embase, and LILACS were searched to identify data on CMV prevalence, seroprevalence, shedding, and transmission rates. The SLR covered the time period of 2010–2020 and focused geographically on Australia, Europe, Israel, Japan, Latin America (LATAM), and North America. Studies were excluded if they were systematic or narrative reviews, abstracts, case series, letters, or correspondence. Studies with sample sizes < 100 were excluded to focus on studies with higher quality of data.

**Results:**

Twenty-nine studies were included. Among adult men, CMV immunoglobulin G (IgG) seroprevalence ranged from 39.3% (France) to 48.0% (United States). Among women of reproductive age in Europe, Japan, LATAM, and North America, CMV IgG seroprevalence was 45.6-95.7%, 60.2%, 58.3-94.5%, and 24.6-81.0%, respectively. Seroprevalence increased with age and was lower in developed than developing countries, but data were limited. No studies of CMV immunoglobulin M (IgM) seroprevalence among men were identified. Among women of reproductive age, CMV IgM seroprevalence was heterogenous across Europe (1.0-4.6%), North America (2.3-4.5%), Japan (0.8%), and LATAM (0-0.7%). CMV seroprevalence correlated with race, ethnicity, socioeconomic status, and education level. CMV shedding ranged between 0% and 70.2% depending on age group. No findings on CMV transmission rates were identified.

**Conclusions:**

Certain populations and regions are at a substantially higher risk of CMV infection. The extensive epidemiologic burden of CMV calls for increased efforts in the research and development of vaccines and treatments.

**Trial registration:**

N/A.

**Supplementary Information:**

The online version contains supplementary material available at 10.1186/s12889-022-13971-7.

## Background

Cytomegalovirus (CMV), a member of the herpesvirus family (*Herpesviridae*), is a pathogen common worldwide that infects a substantial number of individuals at some point in their lives [1]. In the United States, it is estimated that the virus will infect approximately 30% of children by 5 years of age and more than 50% of adults by 40 years of age [2]. Generally, CMV seroprevalence is higher among women, those in older age groups, persons of lower socioeconomic status, and in developing countries [3]. Among women of reproductive age in particular, global CMV seroprevalence ranges from 45 to 100% [3].

CMV can be transmitted through contact with infectious bodily fluids such as blood, saliva, urine, tears, seminal fluid, cervical secretions, and breast milk. In addition, infection is possible following solid organ and stem cell transplantation [2], with CMV representing the most common opportunistic infection among solid organ transplant recipients [4]. After initial CMV infection in a previously seronegative individual (primary infection), reactivation of persistent latent virus or infection with a different CMV strain (nonprimary infection) can occur.

In healthy individuals, CMV infection is typically asymptomatic or causes mild illness [5]; however, CMV transmission from a pregnant woman to her fetus in utero may cause congenital CMV (cCMV), which can result in serious long-term sequelae or death [6–13]. Acquisition of primary CMV infection during pregnancy poses the greatest risk to infants; approximately a third of infants born to mothers with primary CMV infection during pregnancy have cCMV infection [14, 15]. Serious CMV-related sequelae can also occur in those with compromised immune systems, including solid organ or stem cell transplant recipients, individuals on immunosuppressive therapy, or those infected with human immunodeficiency virus (HIV) [16]. CMV is the most common cause of vision loss in individuals with HIV, even while on highly active antiretroviral therapy [17]. Currently, treatments for CMV are limited and no vaccine is available [18]. Thus, development of a CMV vaccine to prevent infection remains a high public health priority.

Given that CMV infection is common globally yet has a variable clinical course and a potential for long-term sequalae, a greater understanding of CMV epidemiologic data worldwide is needed, which can support the development of CMV vaccines and justify vaccine introduction into immunization schedules. Previously conducted systematic literature reviews (SLRs) on CMV prevalence/seroprevalence [1, 19–22], transmission rate [23, 24], or long-term sequelae [7, 25, 26] have been published; however, these SLRs included historical data, and thus, more current estimates of CMV burden are warranted. A thorough understanding of the epidemiologic impact of CMV is also hampered by the variation of burden between countries, within countries, and within subpopulations [1, 3, 24]. Therefore, there is a need to highlight seroprevalence, shedding, and transmission in specific populations affected by CMV. Here, we performed a SLR to describe the most recent (2010–2020) epidemiologic data on CMV seroprevalence, shedding, and transmission across several countries/regions according to population characteristics such as sex, age, at-risk status, socioeconomic status, educational level, and race/ethnicity.

## Methods

**Fig. 1:**
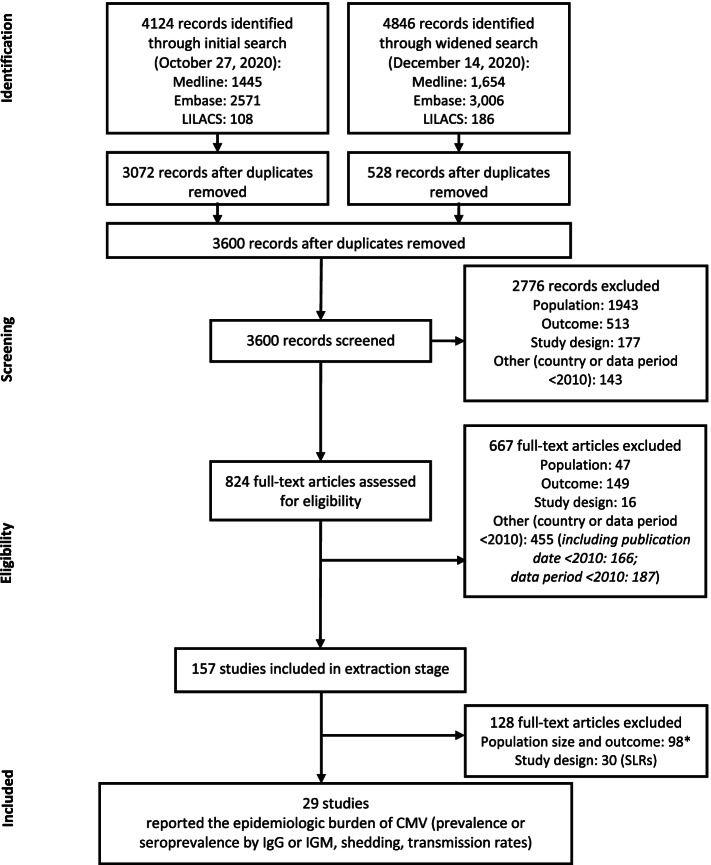
Flow diagram of screening process. *Reasons for exclusion: population size < 100; prevalence or seroprevalence based on a non-IgM or -IgG diagnostic method (eg, reverse transcriptase polymerase chain reaction); or data out of scope of review (incidence, infection rate, mortality, or long-term sequelae). CMV, cytomegalovirus; IgG, immunoglobulin G; IgM, immunoglobulin M; SLR, systematic literature review

A systematic review of the epidemiologic burden of CMV was conducted based on peer-reviewed articles published in the Medline, Embase, and Latin American and Caribbean Health Sciences Literature (LILACS) databases from the year 2000 through December 14, 2020 (an initial search was performed on October 27, 2020, and a widened search with supplemental search terms and additional outcomes of interest was performed on December 14, 2020; Fig. 1; Supplemental Tables 1–6 in Additional File 1). Our search strategy consisted of subject headings (ie, medical subject header [MeSH] and Emtree), keywords, free text terms, and their synonyms and was adapted to the requirements of each queried database (detailed in Supplemental Tables 1–6 in Additional File 1). Medline and Embase were searched for the following themes: ((CMV / cytomegalovirus infections) AND epidemiology AND (epidemiologic studies AND Countries) OR (systematic reviews / meta-analysis)); in LILACS, the search was restricted to CMV AND epidemiology. Each record was assessed for relevance against predefined eligibility criteria (Supplemental Tables 7–8 in Additional File 1). Double independent record selection was performed during title/abstract and full text screening. Discrepancies concerning inclusion or exclusion were resolved after discussion between reviewers or through reconciliation by a third reviewer.

**Table 1:** Categorization of data with definitions

Category	Definition
Age	Newborn: up to 1 month Infants: 2 months to 2 years Children: 3–10 years Adolescent: 11–18 years Adults: ≥ 19 years Elderly: ≥ 60 years
At-risk population	Populations at risk [27]: 1. Individuals with primary immunodeficiencies 2. Individuals with secondary immunodeficiencies caused by disease of the immune system: leukemia and other hematologic malignancies, human immunodeficiency virus infection 3. Critically sick intensive care unit patients 4. Recipients of drugs suppressing the immune system: anti-CD52, anti-CD20, anti-CD25, anti-tumor necrosis factor General population: general population (eg, city or country) Not at risk: population without any risk factor
Sex	Male: men Women of reproductive age: defined as pregnant women, mothers of infants, reproductive-age women, women of childbearing age, teenage and adult women (not elderly). The reproductive age might be defined as age range 15 to 49 years [28]. This definition was used in this review, but if the publications included different age ranges for women of childbearing age or pregnant women, these data were included as well
Social status	As defined by authors of included study
Education level	As defined by authors of included study
Race/ethnicity	As defined by authors of included study
Developed or developing country	As categorized by the International Monetary Fund [29]

The extensive search of bibliographic databases covered the time period of 2000–2020 (2017–2020 for conference abstracts) and was restricted to English language and the following countries and regions: Australia, Latin America (LATAM), Canada, Europe, Israel, Japan, United States, and global (international, worldwide). We included all age groups, mothers and infants with HIV, and specific subpopulations or immunocompromised groups. From this extensive search, we then focused on the most recent data and only extracted data from publications with study data between 2010–2020. If a particular article did not provide information on the study period, the year of publication was considered. For the purpose of this report, SLRs, narrative reviews, abstracts, case series, letters, and correspondence were excluded. Studies with sample sizes < 100 were also excluded in order to focus on studies with higher data quality and an adequate sample size for estimating prevalence.

Initial outcomes of interest were CMV infection rate, force of infection (the rate at which susceptible individuals in a population acquire an infectious disease in that population, per unit time [30]), reactivation, prevalence/seroprevalence, incidence, vertical and horizontal transmission, mortality, pregnancy loss, prevalence of shedding, morbidity, and long-term sequelae/effects. From this initially broad set of outcomes, we focused on CMV prevalence, seroprevalence, shedding, and transmission rate. Outcomes were divided into categories based on the elements of the research methodology or subpopulation; Table 1 presents the adopted data categorizations and definitions for age, at-risk population, sex, social status, education level, race/ethnicity, and developed or developing country. Seroprevalence outcomes were evaluated as region-specific seroprevalence of CMV (immunoglobulin G [IgG] or immunoglobulin M [IgM]) according to sex, age, at-risk population, socioeconomic status, education level, and race/ethnicity. At-risk populations were defined as individuals with primary immunodeficiencies, individuals with secondary immunodeficiencies caused by diseases of the immune system, critically sick intensive care unit patients, and recipients of drugs suppressing the immune system (Table 1). Additionally, CMV seroprevalences were assessed within 10-year age increments for men, women of reproductive age, and adults. CMV shedding and transmission outcomes by age categories were also evaluated.

Outcomes were presented as ranges (minimum–maximum). For single measure estimates wherein it was not possible to determine the interval, the confidence interval was provided (if available within the source reference).

## Results

A total of 4124 records were retrieved through the initial search and 4846 records were retrieved through the widened search of the bibliographic databases (Fig. 1). After removal of duplicates, 3600 records remained for screening. Of these, 2766 irrelevant records were excluded, with a total of 824 full-text articles assessed for eligibility. A total of 157 references were included in the data extraction stage; 128 references were subsequently excluded either because they had a population size of < 100, had outcomes not relevant for the purpose of this report, or were SLRs. In total, 29 studies were included in this epidemiology review (Fig. 1; Supplemental Table 9 in Additional File 1).

The included studies covered data from a total of 14 countries: 2 countries from North America (Canada and the United States), 9 countries from Europe (Bosnia and Herzegovina, Croatia, France, Italy, Norway, Poland, Romania, Spain, and the United Kingdom), 2 countries from LATAM (Brazil and Mexico), and 1 country from other regions (Japan). Most studies were from Mexico (*n* = 4), the United States (*n* = 4), Japan (*n* = 3), Poland (*n* = 3), and the United Kingdom (*n* = 3). Recent epidemiologic data (2015 onwards) were reported in 6 studies; 17 studies presented data before 2015, 5 studies had a data period within 2010–2020, and 1 study did not indicate a data period. Further details of the included studies are shown in Supplemental Table 9 in Additional File 1.

## CMV seroprevalence by sex and age group

### Men and women of reproductive age

#### IgG antibodies

**Fig. 2:**
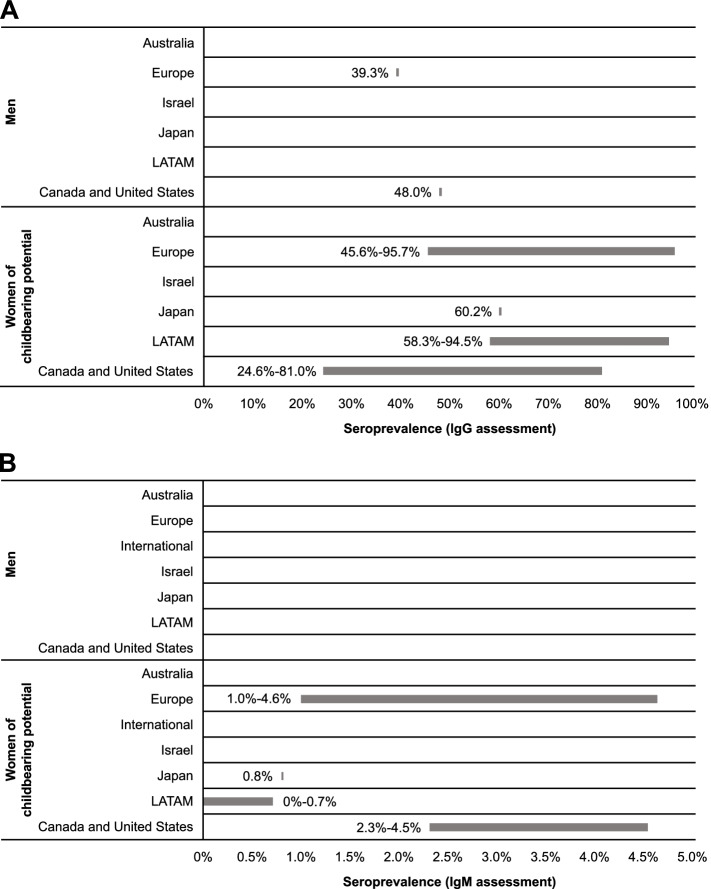
Region-specific cytomegalovirus IgG and IgM seroprevalence among men and women of childbearing potential. IgG, immunoglobulin G; IgM, immunoglobulin M; LATAM, Latin America

The presence of CMV IgG antibodies in the absence of IgM antibodies indicates previous, but not acute, infection [31]. Two studies reported CMV IgG seroprevalence specifically for male populations (Table 2; Fig. 2): 39.3% (95% CI: 34.9-43.8%) in a cross-sectional survey from a nationally representative population-based sample from France (Europe) and 48.0% in a US-based study that utilized a cross-sectional serosurvey among adult residents in North Carolina [32, 33].

**Table 2:** Region-specific CMV seroprevalence (according to sex and age) and CMV shedding (according to age)

	**Australia**	**Europe**	**Israel**	**Japan**	**Latin America**	**Canada and the United States**	**Total**
**Seroprevalence of CMV IgG by sex, % (95% CI**^a^**)**							
Men [32, 33]	NR	39.3 (34.9–43.8)	NR	NR	NR	48.0^b^	39.3–48.0
Women of reproductive age [32, 34–47]	NR	45.6–95.7	NR	60.2^b^	58.3–94.5	24.6–81.0	24.6–95.7
Aged 13–20 years^c^ [37, 44]	NR	94.6^b^	NR	NR	86.5^b^	NR	86.5–94.6
Aged 20–30 years^c^ [36, 37, 41, 44, 45]	NR	58.5–94.9	NR	NR	91.3^b^	54.4^b^	54.4–94.9
Aged 30–40 years^c^ [36, 37, 40, 41, 45–47]	NR	62.3–95.7	NR	NR	NR	40.0–59.7%	40.0–95.7
Aged 40–50 years^c^ [40, 45]	NR	87.7^b^	NR	NR	NR	69.8^b^	69.8–87.7
**Seroprevalence of CMV IgM by sex, % (95% CI**^a^**)**							
Men^d^	NR	NR	NR	NR	NR	NR	NR
Women of reproductive age [34, 36, 41–45]	NR	1.0–4.6	NR	0.8^b^	0.0–0.7	2.3–4.5	0.0–4.6
Aged 20–30 years^c^ [41, 45]	NR	1.0–2.0	NR	NR	NR	4.5^b^	1.0–2.0
Aged 30–40 years^c^ [41, 45]	NR	2.8^b^	NR	NR	NR	2.3^b^	2.3–2.8
Aged 40–50 years^c^ [45]	NR	NR	NR	NR	NR	2.4^b^	2.4
**Seroprevalence of CMV IgG by age, % (95% CI**^a^**)**							
Adults (≥ 19 years) [32, 33, 35–37, 40–42, 44–55]	NR	44.4–95.7	NR	67.2–70.9	59.1–91.3	33.0–81.0	33.0–95.7
Elderly (≥ 60 years) [35, 51, 56, 57]	NR	64.5–97.7	NR	NR	NR	NR	64.5–97.7
Age intervals							
12–20 years^c^ [35, 37, 44, 45]	NR	70.3–94.6	NR	NR	86.5^b^	47.3^b^	47.3–94.6
20–30 years^c^ [36, 37, 41, 44, 45, 51]	NR	58.5–94.9	NR	NR	91.3^b^	54.4^b^	54.4–94.9
30–40 years^c^ [33, 36, 37, 40, 41, 45, 46, 51]	NR	62.3–95.7	NR	NR	NR	40.0–59.7	40.0–95.7
40–50 years^c^ [33, 40, 45, 51]	NR	85.3–87.7	NR	NR	NR	67.0–69.8	67.0–87.7
50–60 years^c^ [33, 51]	NR	91.5 (87.8–94.4)	NR	NR	NR	61.0^b^	61.0–91.5
60–100 years^c^ [35, 51, 56, 57]	NR	64.5–97.7	NR	NR	NR	NR	64.5–97.7
**Seroprevalence of CMV IgM by age, % (95% CI**^a^**)**							
Adults (≥ 19 years) [41, 45, 51]	NR	1.0–6.7	NR	NR	NR	2.3–4.5	1.0–6.7
Elderly (≥ 60 years) [51]	NR	3.5 (1.7–6.3)	NR	NR	NR	NR	3.5 (1.7–6.3)
Age intervals							
12–19 years^c^ [45]	NR	NR	NR	NR	NR	2.6^b^	2.6
19–30 years^c^ [41, 45, 51]	NR	1.0–6.7	NR	NR	NR	4.5^b^	1.0–6.7
30–40 years^c^ [41, 45, 51]	NR	2.4–2.8	NR	NR	NR	2.3^b^	2.3–2.8
40–50 years^c^ [45, 51]	NR	4.3 (2.3–7.4)	NR	NR	NR	2.4^b^	2.4–4.3
50–60 years^c^ [51]	NR	4.3 (2.3–7.2)	NR	NR	NR	NR	4.3 (2.3–7.2)
60–100 years^c^ [51]	NR	3.5 (1.7–6.3)	NR	NR	NR	NR	3.5 (1.7–6.3)
**CMV shedding by age (regardless of diagnostic method), % (95% CI**^**a**^**)**							
Newborns/infants [48]	NR	0.0^b^	NR	NR	NR	NR	0.0^b^
Newborns/infants to adolescents	NR	NR	NR	NR	NR	NR	NR
Newborns/infants to children [48, 58, 59]	NR	11.0–51.9	NR	NR	NR	17.0^b^	11.0–51.9
Children [48]	NR	5.2^b^	NR	NR	NR	NR	5.2^b^
Adolescents [48]	NR	0.0^b^	NR	NR	NR	NR	0.0^b^
Adolescents to adults [60]	NR	70.2^b^	NR	NR	NR	NR	70.2^b^

*CI* Confidence interval, *CMV* Cytomegalovirus, *IgG* Immunoglobulin G, *IgM* Immunoglobulin M, *NR* Not reported

^a^Single measure estimates are presented with 95% CIs (if available in primary publication)

^b^Confidence intervals were not reported in primary publication

^c^Age ranges with single-year overlap were empirically determined to account for variable age ranges and lack of single-year data in primary publications

^d^Data for 10-year-old age groups have not been identified

Comparatively, 15 studies from Japan, Europe, LATAM, Canada, and the United States reported CMV IgG seroprevalence estimates for women of reproductive age (Table 2; Fig. 2). In Japan, seroprevalence was estimated as 60.2% [34]. In Europe and LATAM, seroprevalence was similar across studies, ranging from 45.6 to 95.7% in Europe [32, 35–41] and 58.3 to 94.5% in LATAM [42–44]. In North America, seroprevalence ranged from 24.6 to 81.0% [45–47].

No data were found for CMV IgG seroprevalence across age categories in men. Seroprevalence among women of reproductive age suggests a potential increase with age; however, these findings are limited by the small dataset. Seroprevalence of CMV infection in pregnant women in Mexico was higher in those aged 20 to 30 years than those aged ≤ 20 years (91.3 vs 86.5%, respectively) [44]. Studies from Canada and the United States indicate that seroprevalence was higher among women aged > 40 years compared with women aged ≤ 40 years [45–47]. No noticeable age-related trends were identified among European studies [36, 37, 40, 41].

In developing countries of the European and LATAM regions included in this report, reported CMV IgG seroprevalences among women of reproductive age were similar. Studies conducted in Mexico reported CMV IgG seroprevalences of 58.3 to 94.5% for women of reproductive age [37, 42–44]. In Europe, CMV IgG seroprevalence ranged from 57.3% among women of reproductive age in Poland [40] to 95.7% in Romania [37]. In comparison, in developed countries of Europe, CMV IgG seroprevalence among women of reproductive age ranged between 45.6 and 65.9% [32, 36, 39].

#### IgM antibodies

The presence of CMV IgM antibodies may be indicative of recent infection (ie, primary, reactivation, or reinfection) [31]. No studies with data on CMV IgM seroprevalence among men were identified. Among women of reproductive age, estimates suggest burden of primary and secondary CMV infection was similar in Europe (1.0-4.6%) [36, 41] and North America (2.3-4.5%) [45]; these seroprevalences were higher than those observed in Japan (0.8%) [34] and LATAM (0-0.7%) [42–44] (Table 2; Fig. 2). While regional differences in CMV seroprevalence have historically been documented, the small number of studies in this SLR showed seroprevalence to be heterogenous with regional patterns difficult to discern.

### Adults and the elderly

#### IgG antibodies

**Fig. 3:**
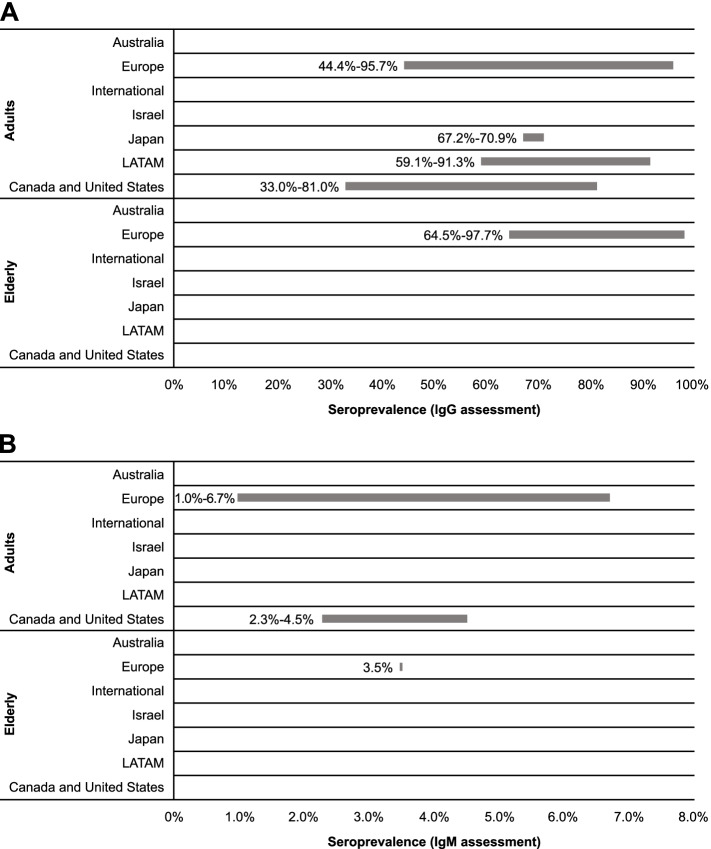
Region-specific cytomegalovirus IgG and IgM seroprevalence among adults and the elderly. IgG, immunoglobulin G; IgM, immunoglobulin M; LATAM, Latin America

Twenty-two references were included for assessing seroprevalence across age categories based on detection of CMV IgG antibodies (Table 2). These articles reported data for general populations, healthy or immunocompetent populations (ie, without specific diseases and otherwise healthy), and adults. Among adults, seroprevalence ranged most broadly in European countries (44.4-95.7%) [32, 35–37, 40, 41, 48–51], with the narrowest range observed for Japanese studies (67.2-70.9%; Fig. 3) [52, 53]. LATAM and North America had notable differences in seroprevalence, with ranges of 59.1 to 91.3% [42, 44, 54, 55] and 33.0 to 81.0% [33, 45–47], respectively. Comparing the range maximums, Europe appeared to have the highest seroprevalence of CMV among adults. Similarly, among the elderly in Europe, multiple articles indicated approximately 2% of the population is seronegative for CMV IgG, suggesting a high seroprevalence of CMV among the elderly in this region [35, 51, 56, 57].

Recent data reported within the identified studies suggested differences in seroprevalence ranges across age categories (Table 2). Across European studies, the maximum values of the ranges did not substantially vary, but the minimum values increased with age intervals. Data from LATAM indicated that seroprevalence was higher in the 20- to 30-year age group in comparison to the 12- to 20-year age group (91.3 vs 86.5%) [44]. Age-related increases in seroprevalence were also noticeable in North American studies [33, 45, 46].

CMV IgG seroprevalence was higher among developing than developed countries included in this report. Among adults, CMV IgG seroprevalence ranged from 33.0 to 81.0% for developed countries [32, 33, 36, 45–50, 52, 53] and 59.1 to 95.7% for developing countries [35, 37, 40, 42, 44, 51, 54, 55]. For the elderly, seroprevalence was 64.5 to 96.2% for developed countries [56, 57] and 93.8 to 97.7% for developing countries [35, 51].

#### IgM antibodies

We identified 3 studies published since 2010 that provided data on CMV IgM seroprevalence in various adult age categories in general populations; within these studies, no clear regional trend was observed (Table 2; Fig. 3). Across European studies, CMV IgM seroprevalence was reported as 1.0 to 6.7% for adults [41, 51] and 3.5% (95% CI, 1.7-6.3%) for elderly populations [51]. Within the US study identified, CMV IgM seroprevalence among adults was reported as 2.3 to 4.5% [45].

Overall, CMV IgM seroprevalence was similar for developing and developed countries. Seroprevalence of CMV IgM was estimated as 2.3 to 4.5% among adults in developed countries [45] and 1.0 to 6.7% in developing countries [41, 51]. Among the elderly, only data for developing countries were available, with a seroprevalence of 3.5% (95% CI, 1.7-6.3%) reported for the elderly population in Croatia [51].

A total of 3 publications provided data on both IgM and IgG seroprevalences. In a Polish population of pregnant women aged 16 to 45 years, IgM seroprevalence was 2.2% and IgG seroprevalence was 62.4% [41]. In US women 12 to 49 years of age, the seroprevalence of CMV IgM and IgG was 3.0% and 57.9%, respectively [45]. CMV IgG seroprevalence generally increased with age, whereas IgM seroprevalence did not show a clear age-related trend in these populations; however, the correlations between age and IgM or IgG seroprevalence were not statistically analyzed [45]. The study from Croatia reported that among the general population (aged 1 month to 82 years), the seroprevalence of IgM and IgG was 4.3 and 74.4%, respectively; among the elderly, seroprevalence was 3.5 and 93.8%, respectively [51]. Neither the Croatian nor US studies assessed the statistical correlations between IgM antibody titers and IgG antibody titers [45, 51].

As CMV IgM is not a precise indicator of primary versus nonprimary CMV infection, the presence of low CMV IgG avidity can be a useful serologic indicator of primary CMV infection. One study from the United States provided data on the low CMV IgG avidity in the context of CMV IgM prevalence, with the authors stating that primary CMV infection was estimated in 14 to 18% of CMV IgM-positive women, as they had low IgG avidity [45]. While IgM was not correlated with age, the prevalence of low CMV IgG avidity decreased with age [45].

## CMV seroprevalence by risk factors

### At-risk populations

We identified only 1 article published within the last decade (2010–2020) among at-risk populations [51] (Table 3), which was defined as critically sick intensive care unit patients, those with primary immunodeficiencies, those with secondary immunodeficiencies caused by disease of the immune system, and recipients of immunosuppressing drugs. During a 3-year period (2013–2015), serum samples were collected from Croatian (Europe) residents (of any age) and screened for CMV IgM and IgG antibodies. Among hemodialysis patients, hemodialysis was the main predictor for CMV IgG seropositivity, with CMV seroprevalences reported at 91.4% (95% Cl, 87.7-94.2%) [51]. Interestingly, CMV reactivation/reinfection was most common in this population (92.3%). Overall, these reported seroprevalences among hemodialysis patients appeared higher than those estimates across European adult populations (44.4-95.7%; Table 2). CMV IgM seropositivity seroprevalences were reported as 8.6% (95% CI, 5.7-12.3%) [51].

**Table 3:** Region-specific CMV seroprevalence according to population at risk, socioeconomic status, education level, and race/ethnicity

	**Australia**	**Europe**	**Israel**	**Japan**	**Latin America**	**Canada and the United States**	**Total**
**Seroprevalence of CMV IgG by population at risk, % (95% CI**^a^**)**							
Population at risk [51]	NR	91.4 (87.7–94.2)	NR	NR	NR	NR	91.4 (87.7–94.2)
**Seroprevalence of CMV IgM by population at risk, % (95% CI**^a^**)**							
Population at risk [51]	NR	8.6 (5.7–12.3)	NR	NR	NR	NR	8.6 (5.7–12.3)
**Seroprevalence of CMV IgG by socioeconomic status, % (95% CI**^a^**)**							
Household income, Canadian dollars [46]							
0–59,999	NR	NR	NR	NR	NR	58.5^b^	58.5
60,000–99,999	NR	NR	NR	NR	NR	34.5^b^	34.5
≥ 100,000	NR	NR	NR	NR	NR	27.1^b^	27.1
Family income to poverty ratio [61]							
Below poverty level	NR	NR	NR	NR	NR	26.4–31.1	26.4–31.1
At or above poverty level	NR	NR	NR	NR	NR	14.9–27.6	14.9–27.6
Family income^c^ [47]							
Low	NR	NR	NR	NR	NR	81.0^b^	81.0
Middle class	NR	NR	NR	NR	NR	54.0^b^	54.0
High	NR	NR	NR	NR	NR	35.0^b^	35.0
Financial status^d^ [41]							
Average	NR	63.5^b^	NR	NR	NR	NR	63.5^b^
Good	NR	63.5^b^	NR	NR	NR	NR	63.5^b^
Unknown	NR	60.9^b^	NR	NR	NR	NR	60.9^b^
**Seroprevalence of CMV IgM by socioeconomic status, % (95% CI**^a^**)**							
Financial status^e^ [41]							
Average	NR	1.4^b^	NR	NR	NR	NR	1.4^b^
Good	NR	2.6^b^	NR	NR	NR	NR	2.6^b^
Unknown	NR	2.6^b^	NR	NR	NR	NR	2.6^b^
**Seroprevalence of CMV IgG by education level, % (95% CI**^a^**)**							
Household reference person’s education level [61]							
Less than high school diploma	NR	NR	NR	NR	NR	31.3–37.2	31.3–37.2
High school diploma, GED, associate degree, some college	NR	NR	NR	NR	NR	16.7–22.6	16.7–22.6
College degree or higher	NR	NR	NR	NR	NR	17.8–34.7	17.8–34.7
Education level							
Up to university [47]	NR	NR	NR	NR	NR	60.0^b^	60.0^b^
University [47]	NR	NR	NR	NR	NR	51.0^b^	51.0^b^
Higher [41]	NR	58.0^b^	NR	NR	NR	NR	58.0^b^
Secondary [41]	NR	64.5^b^	NR	NR	NR	NR	64.5^b^
Primary and vocational [41]	NR	72.9^b^	NR	NR	NR	NR	72.9^b^
**Seroprevalence of CMV IgM by education level, % (95% CI**^a^**)**							
Education level [41]							
Higher	NR	2.1^b^	NR	NR	NR	NR	2.1^b^
Secondary	NR	1.9^b^	NR	NR	NR	NR	1.9^b^
Primary and vocational	NR	2.1^b^	NR	NR	NR	NR	2.1^b^
**Seroprevalence of CMV IgG by race/ethnicity, % (95% CI**^a^**)**							
White (99.3%)^f^ [57]	NR	96.2^b^	NR	NR	NR	NR	96.2^b^
Mestizo (98.9%)/White (1.1%) [44]	NR	NR	NR	NR	89.6^b^	NR	89.6^b^
Hispanic [61]	NR	NR	NR	NR	NR	31.0–36.7	31.0–36.7
Non-Hispanic White [61]	NR	NR	NR	NR	NR	10.6–24.2	10.6–24.2
Non-Hispanic Black [61]	NR	NR	NR	NR	NR	15.9–24.6	15.9–24.6
Non-Hispanic other/multiracial [61]	NR	NR	NR	NR	NR	37.0–40.0	37.0–40.0

*CI* Confidence interval, *CMV* Cytomegalovirus, *GED* General educational development, *IgG* Immunoglobulin G, *IgM* Immunoglobulin M, *NR* Not reported

^a^Single measure estimates are presented with 95% CIs (if available in primary publication)

^b^Confidence intervals were not reported in primary publication

^c^Family income was defined in the primary publication as low: ≤ $30,000/year; middle: $31,000-$99,000/year; high: ≥ $100,000/year [47]

^d^Details of the definition for financial status were not provided in the primary publication [41]. The following data were not included in our review due to population sizes < 100: those with financial statuses of bad (*n* = 48; seroprevalence, 58.30%) and very good (*n* = 76; seroprevalence, 53.90%)

^e^Details of the definition for financial status were not provided in the primary publication [41]. The following data were not included in our review due to population sizes < 100: those with financial status of bad (*n* = 48; seroprevalence, 2.1%) and very good (*n* = 76; seroprevalence, 2.6%)

^f^The primary publication [57] indicated that 99.3% of case patients were White

### Socioeconomic status

Recent studies from North America have evaluated the relationship between CMV seroprevalence and household income and poverty level (Table 3). A study from Canada indicated that CMV IgG seroprevalence among a cohort of pregnant women was 58.5%, 34.5%, and 27.1% for household incomes of $0 to $59,999, $60,000 to $99,999, and ≥ $100,000 (Canadian dollars), respectively [46]. In a separate single-center study in Canada of women from low-, middle-, and high-income families, CMV IgG seroprevalence was 81.0%, 54.0%, and 35.0%, respectively [47]. In a study among US children aged 1 to 5 years during 2011–2012, CMV IgG seroprevalence was 2-times higher among children from households with a family income to poverty ratio below the poverty level (< 1.0) than those from households above the poverty level (≥ 1.0; 31.1 vs 14.9%, respectively) [61]; however, this trend was not observed in 2017–2018 (26.4 vs 27.6%, respectively) [61]. This lack of observable difference likely reflects an increase in CMV seroprevalence among children at or above the poverty level from 2011–2012 to 2017–2018. In Poland, CMV IgG and IgM seroprevalences did not differ significantly by financial status [41].

### Education level

In a Canadian study of pregnant women, CMV IgG seroprevalence was 60.0% among adults with a non-university education level and 51.0% among adults with a university education level [47] (Table 3). When evaluating associations between education level groups, non-university educated women were more likely to be CMV IgG seropositive than university educated women (OR, 2.43; 95% CI, 1.37–4.32) [47]. Similar results were reported for a cohort of pregnant Polish (European) adult women, where CMV IgG prevalence was evaluated according to education level; seroprevalence was 58.0% among women with higher education, 64.5% among women with secondary education, and 72.9% among women with primary/vocational education [41]. Interestingly, there was no descriptive or inferential trend observed for evaluations using CMV IgM positive serologic data (2.1% among adults with higher education, 1.9% among adults with secondary education, 2.1% among adults with primary/vocational education) [41]. These results were likely due to the small sample size of CMV IgM seropositive women (*n* = 25). Among US children aged 1 to 5 years, the range of CMV IgG seroprevalence was reported as higher for households with survey participants whose education level was less than a high school diploma (31.3%) versus those households with participants with a high school diploma and some college education (16.7%) or with a college degree or more (17.8%) from 2011–2012 [61]. Among households with lower education levels, CMV IgG seroprevalence did not significantly increase from 2011–2012 to 2017–2018 (prevalence difference of 5.9 points); however, a substantial increase in seroprevalence during this time frame was observed for households with individuals with a college degree or more (prevalence difference of 16.8) [61].

### Race and ethnicity

Our review identified recent articles from Spain (Europe), Mexico (LATAM), and the United States that explored the relationship between CMV IgG seroprevalence and race/ethnicity (Table 3). In a single-center case control study in Spain among a predominantly White study population, CMV IgG seroprevalence among elderly patients was reported as 96.2% [57]. In a cross-sectional study of pregnant women in Mexico, CMV IgG seroprevalence was 89.6% in a population predominantly of Mestizo ethnic descent [44]. From these studies, it is difficult to confirm the role of ethnicity as a risk factor for CMV infection. Utilizing the National Health and Nutrition Examination Survey data collected in the United States during 2011–2012, CMV IgG seroprevalence by race/ethnicity of children 1 to 5 years of age were reported as 37.0% among non-Hispanic other/multiracial, 31.0% among Hispanic, 15.9% among Non-Hispanic Black, and 10.6% among non-Hispanic White ethnicities [61]. When comparing to CMV IgG seroprevalence estimates from 2017–2018, an increase was observed across each race/ethnicity category, with a notable difference among non-Hispanic White children (10.6-24.2%) [61]. The only exception was a decrease in seroprevalence observed in Non-Hispanic Black children (24.6-15.9%).

## CMV shedding and transmission

No studies reporting data for CMV transmission rate were identified. Between 2010 and 2020, only 4 studies provided data on the prevalence of CMV shedding, which were from developed countries (England, France, Spain, and the United States; Table 2). Studying urine samples in the British population, shedding was reported as 0% among newborns and infants aged < 2 weeks, 11.0% among those aged 2 weeks to 5 years, 5.2% among those aged 6 to 10 years, and 0% among adolescents (aged 11–15 years) [48]. In the United States, shedding was reported as 17.0% among children aged 0 to 47 months [58]. In a feasibility study conducted in French daycare centers among children aged 3 months to 6 years, saliva specimens confirmed CMV shedding in 51.9% of sites [59]. In a study conducted in the United States, which utilized saliva as well as urine specimens from children aged 0 to 47 months, half of seropositive children were shedding CMV in at least 1 fluid [58]. Breast milk was screened for CMV DNA in a prospective Spanish study, which found 70.2% of specimens positive for CMV [60].

## Discussion

This SLR aimed to provide an updated understanding of the current epidemiology of CMV, including prevalence/seroprevalence, shedding, and transmission, across regions and subpopulations. Compiling and assessing these datasets highlights the current knowledge gaps and may aid in guiding policy decisions within the healthcare sector, including those related to CMV clinical guidelines, screening, and potential future treatment and prevention options.

A total of 29 studies were included in our review, with the majority reported from Europe and North America (Canada and the United States). Among women of reproductive age, CMV IgG seroprevalence ranged from 24.6 to 95.7%, which was generally in line with estimates from a recent meta-regression analysis that estimated the global CMV seroprevalence among women of reproductive age as 86%, with the highest seroprevalence observed in the World Health Organization (WHO) Eastern Mediterranean region (92%; 95% uncertainty interval [UI], 88-95%) and the lowest seroprevalence observed in the WHO European region (70%; 95% UI, 63-76%) [1]. However, our observed ranges were wider than the uncertainty range in this prior report. Among men, we identified only 2 studies reporting CMV IgG seroprevalence and no studies reporting CMV IgM seroprevalence. Thus, similar to reports before 2010, seroprevalence studies continue to primarily focus on women [1, 3, 62]. Overall, our available data indicated that CMV seroprevalence was higher among women of reproductive age than men, in agreement with a prior systematic review from 2010 [3]. Childcare is generally believed to contribute to higher seroprevalence among women [41, 63–65].

Our review also evaluated seroprevalence using IgG and IgM diagnostic methods. CMV IgM antibodies can be used as a marker for primary CMV infection and reactivation/reoccurrence or reinfection (nonprimary infection) and as a potential marker for prevalence of transmission at the time of testing [66]. In studies utilizing IgM as a diagnostic method, ranges for prevalence were narrower and lower compared with those utilizing IgG methods. This result is expected, as IgM production occurs first after CMV infection, while IgG levels begin to rise several weeks after infection and remain in the blood throughout a person’s lifetime. Therefore, outcomes based on IgM may be more representative of new and active infections, whereas IgG would indicate the overall number of infected patients. However, CMV IgM antibodies can also be associated with both primary and nonprimary CMV infection; thus, distinguishing primary CMV infection requires the detection of low CMV IgG avidity. One study from the United States reported low CMV IgG avidity estimates in 14% to 18% of CMV IgM-positive women, suggesting primary CMV infection [45]. Limitations of IgM assays should be considered when interpreting IgM data reported throughout this SLR; variation exists between IgM diagnostic assays, indicating assays are less standardized and therefore potentially less reliable than assays for anti-CMV IgG [67]. In addition, there is a risk that CMV IgM assays may be confounded by antibody cross-reactivity, for example, to Epstein Barr virus [68].

Previous research has implicated socioeconomic disparities, race, and ethnicity as risk factors of CMV infection and disease [69–72]. Our review also indicates an association between CMV seroprevalence and education level, social status, household income, and race and ethnicity [3, 32, 38, 41, 42, 44, 46, 47, 55, 58, 61]. This potential association may be based on lifestyle, population density, sexual activity, number of children per family, and child-rearing practices that may be rooted in culture or economics (ie, frequency and duration of breastfeeding, childcare arrangements, and customs that increase saliva sharing with young children) [3, 73]. For example, it has been estimated that CMV occurs in 32% of children attending daycare centers worldwide, with a 2.7-times higher chance of CMV positivity among children attending daycare centers [22]. An analytical model also indicated that hygiene education was greatly effective in prevention of poor outcomes related to CMV infection, estimating that hygiene promotion was associated with a 50% risk reduction for fetal infections in CMV-seronegative populations [74]. Overall, additional insight into the epidemiologic burden of CMV across different risk factors is needed, which can help guide targeted strategies for those populations at greatest risk for infection and disease.

Only 4 studies evaluating CMV shedding were identified in our review, which indicated shedding prevalence ranged from 11.0 to 51.9% in newborns to children aged 10 years. Due to the low number of included studies, no definite conclusions about the prevalence of CMV shedding across age groups could be drawn. However, prior findings have indicated that shedding of CMV is more prevalent among younger age groups, particularly those < 2 years of age [75]. Further, no studies on CMV transmission rates were identified in our review. Taken together, our findings underscore the current need for more recent assessments of CMV shedding and transmission.

Our systematic review was strengthened by focusing on the most recent data on CMV epidemiology (2010–2020) and only including studies with sample sizes > 100 to collate data from studies that provide high-quality data and avoid selection bias. However, we did not synthesize data (eg, by meta-analysis) as provided by prior publications [1, 3, 19, 20, 22, 62]. Additionally, statistical comparisons were not included in this report, as any analysis between countries or across time is outside the scope of our systematic review and would require additional analyses to account for the heterogeneity of studies and changes in technology and methodology. Notably, a minimal number of studies from the Asia–Pacific region were identified in our review, which was expected due to our limited review time frame to capture the most current evidence and geographic search restrictions. Additional work is needed to review the CMV seroprevalence literature from China and India, as well as Africa. Further, although permitted within the geographic scope, no studies were identified from Australia or Israel and future research on CMV seroprevalence is warranted in these countries. In the case of the Israel, initial studies identified in our broad search reported prevalence but not CMV IgM or IgG seroprevalence or were congress abstracts and, thus, did not meet the pre-defined inclusion criteria. In addition, although our review suggests CMV seropositivity increased with age and was lower for developed than developing countries (in alignment with previous data [3]), only limited information was available for these comparisons, thereby restricting an in-depth analysis and inferences. Additional studies that evaluate the age-related seroprevalence of CMV are essential, while studies that evaluate CMV epidemiology in the context of developing regions would aid in deciphering this burden and could guide local clinical recommendations and policy-making decisions towards future interventions such as vaccines.

While our systematic review aimed to elucidate the current epidemiologic burden of CMV across global regions and subpopulations, our study also highlights the lack of recent studies investigating the seroprevalence of CMV among key demographics and countries. Overall, the lack of surveillance and existing evidence in the general population limits our understanding of the causal pathways between CMV infection, disease, and clinically diagnosed outcomes that are critical for the healthcare and policy sectors. Although CMV infection is mild in severity or asymptomatic for the majority of the population, individuals who are immunocompromised (including those undergoing transplant surgery) and neonates have a unique risk of severe disease [5] and would benefit from improved options for managing the risk of CMV infection. Currently, treatments for CMV are limited and no vaccines are available to prevent against CMV infection and disease, although multiple candidate vaccines are currently in clinical development [76]. Routine screening of pregnant women for CMV infection is also not recommended or is subject to debate [77], and interventions to reduce the risk of maternal CMV infection are limited to behavioral practices (ie, hand washing, avoiding contact with a young child’s saliva/urine, etc.)[78, 79]. Targeted newborn screening for CMV has been implemented in some states within the United States, as well as some integrated health systems; however, universal routine newborn screening for CMV is not performed globally [80]. Thus, comprehensive CMV epidemiologic studies are imperative toward furthering our understanding of CMV and associated disease, which in turn can guide public health strategies to reduce disease burden in vulnerable populations through screening, treatment, and vaccine development.

## Supplementary Information

**Additional file 1.**

## Data Availability

The data summarized in this review are from published articles and are publicly available.

## Abbreviations

cCMV: Congenital cytomegalovirus
CI: Confidence interval
CMV: Cytomegalovirus
GED: General educational development
HIV: Human immunodeficiency virus
IgG: Immunoglobulin G
IgM: Immunoglobulin M
LATAM: Latin America
MeSH: Medical subject header
NR: Not reported
RT-PCR: Reverse transcriptase polymerase chain reaction
SLR: Systematic literature review
