# Supplementary material for: A systematic literature review of the global seroprevalence of cytomegalovirus: possible implications for treatment, screening, and vaccine development
Source: BMC Public Health. 2022 Sep 1;22:1659. doi: 10.1186/s12889-022-13971-7 (PMC9435408; doi:10.1186/s12889-022-13971-7)
Supplement: Supplementary file 1 — Additional file 1. [file 12889_2022_13971_MOESM1_ESM.pdf]

Supplementary Appendix

1. Supplement 1. Full search strategy

Initial search strategies

Supplemental Table 1. Initial search strategy for Medline (includes Medline in Process, Pubmed Not Medline, In Data Review, Publisher)

	Ovid MEDLINE®
**Search Platform:**	Ovid
**Date of Search:**	October 27, 2020<br><br>[Last Database Update: October 26, 2020]
**Date Range Searched:**	1946 to current, restricted to 2000-2020
**Search Filters:**	In development of the search strategies the following search filters were used and partly modified:<br><br>Systematic Reviews<br><br>- SIGN Search Strategy Systematic Reviews, OVID format. Scottish Intermediate Guidelines Network (SIGN), Filter Systematic Reviews. Available from: http://sign.ac.uk/search-filters.html (Word document last modified April 25, 2017; cited May 13, 2020) [supplemented by additional search terms]<br><br>Country restrictions:<br><br>- Campbell, Sandy. Filter to Retrieve Studies Related to Canada, Canadian Provinces, and the One Hundred Largest Canadian Centres from the OVID MEDLINE Database. John W. Scott Health Sciences Library, University of Alberta. Rev. March 06, 2020. Available from: http://guides.library.ualberta.ca/health-sciences-search-filters/geographic-filters [used, partly modified]

	- UAB Libraries. PubMed via LHL: Hedges: Search filter United States (work in progress!) [Internet], Cited: October 19, 2020. Available from: https://guides.library.uab.edu/pubmed/hedges [used, partly modified]<br>- Ayiku L, Levay P, Hudson T, Craven J, Barrett E, Finnegan A and Adams R. The MEDLINE UK filter: development and validation of a geographic search filter to retrieve research about the UK from OVID MEDLINE. Health Information and Libraries Journal, 2017 34 (3): 200-216. [consulted, partly used /adapted]

#	Search Terms	Hits
1	*Cytomegalovirus/	12937
2	exp *Cytomegalovirus Infections/	19871
3	Cytomegalovir$.kf.	3627
4	Cytomegalovirus/ and (Cytomegalovir$ or cmv or hcmv or ccmv or human herpesvirus 5 or human herpes virus 5 or human herpesvirus type 5 or human herpes virus type 5 or human betaherpesvirus 5 or human beta-herpesvirus 5 or HHV 5 or HHV5 or salivary gland virus$ or cytomegalic).ab.	16581
5	exp Cytomegalovirus Infections/ and (Cytomegalovir$ or cmv or hcmv or ccmv or human herpesvirus 5 or human herpes virus 5 or human herpesvirus type 5 or human herpes virus type 5 or human betaherpesvirus 5 or human beta-herpesvirus 5 or HHV 5 or HHV5 or salivary gland virus$ or cytomegalic).ab.	17633
6	(Cytomegalovir$ or cmv or hcmv or ccmv or human herpesvirus 5 or human herpes virus 5 or human herpesvirus type 5 or human herpes virus type 5 or human betaherpesvirus 5 or human beta-herpesvirus 5 or HHV 5 or HHV5 or salivary gland virus$).ti,kf.	27806
7	Cytomegalovir$.ab. /freq=2	4967
8	Cytomegalovir$.af. and (cmv or hcmv or ccmv or hhv 5 or hhv5).ab. /freq=2	20759

9	(Cytomegalovir$ or cmv or hcmv or ccmv or human herpesvirus 5 or human herpes virus 5 or human herpesvirus type 5 or human herpes virus type 5 or human betaherpesvirus 5 or human beta-herpesvirus 5 or HHV 5 or HHV5 or salivary gland virus$).ab. and ("in data review" or in process or publisher or "pubmed not medline").st.	3802
10	or/1-9	39613
11	exp Cytomegalovirus Infections/ep, mo	3363
12	Virus Diseases/ep, mo	4520
13	Infant, Newborn, Diseases/ep, mo	4524
14	Infant, Premature, Diseases/ep, mo	3652
15	Pregnancy Complications, Infectious/ep, mo, sn	7879
16	Infectious Disease Transmission, Vertical/sn	1145
17	Disease Transmission, Infectious/sn	753
18	exp prenatal diagnosis/sn	2026
19	Prevalence/	296299
20	Incidence/	266173
21	Mortality/ or Child Mortality/ or Fetal Mortality/ or Hospital Mortality.mp. or exp Infant Mortality/ or mortality, premature/ [mp=title, abstract, original title, name of substance word, subject heading word, floating sub-heading word, keyword heading word, organism supplementary concept word, protocol supplementary concept word, rare disease supplementary concept word, unique identifier, synonyms]	133712
22	Seroepidemiologic Studies/	20786
23	Neonatal Screening/	10284
24	dried blood spot testing/	1516
25	Virus shedding/	3452

26	(prevalence$ or prevalent or incidence$ or incident or mortalit$ or epidemiolog$ or seroepidemiolog$ or seroprevalen$ or seroinciden$ or serosurvey$ or sero-survey$ or serosurveillance or sero surveillance or morbidity or seropositiv$).ti,ab,kf.	2581314
27	(cases adj4 (birth$ or year$ or positive or symptomatic or asymptomatic)).ti,ab.	93518
28	(CMV cases or HCMV cases or CCMV cases).ti,ab.	50
29	((transmission or diagnosis) adj4 rate$).ti,ab.	17115
30	(transmission adj3 risk).mp.	12947
31	(infection rate$ or disease rate$).ti,ab,kf.	26954
32	Frequency.ti,ab.	836562
33	((neonat$ or newborn$ or prenatal or maternal) adj2 screening).ti,ab.	15905
34	Shedding.ti,ab,kf.	26093
35	or/11-34	3550877
36	Epidemiologic studies/	8440
37	exp case control studies/	1113112
38	exp cohort studies/	2046004
39	Retrospective Studies/	846708
40	Cross-sectional studies/	341206
41	observational studies as topic/ or observational study/	92324
42	evaluation studies/	254447
43	Databases, Factual/ or Data Collection/	171169
44	(Long-term or observational or cohort? or longitudinal or prospective or retrospective or comparative or case-control or cross-sectional or survey$).ti.	935483
45	(real-world or real life).ti.	14230

46	(cohort analys$ or cohort stud$ or longitudinal stud$ or chart review$ or medical record review$ or observational stud$ or retrospective analys$ or retrospective chart review? or retrospective clinical stud$ or retrospective cohort$ or retrospective stud$ or retrospective database$ or retrospective observational$ or cross-sectional stud$ or cross-sectional setting$ or case control stud$ or case control setting or nested case control stud$ or matched case-control stud$).kf.	24977
47	(cohort adj (study or studies or analy$)).ti,ab.	222847
48	(cohort$ adj2 (birth$ or newborn$)).ti,ab,kw.	18603
49	((longitudinal or cross sectional or case-control or pragmatic or large scale) adj (study or studies)).ti,ab.	356940
50	((observational or retrospective or prospective) adj3 (study or studies or cohort$1 or data$ or analys#s)).ti,ab.	870211
51	((real world or real life) adj3 (data$ or setting or cohort$ or stud$ or experience$ or result$)).ti,ab.	21155
52	(data adj (report$ or data system$1)).ti,ab.	11511
53	Electronic health records/	20420
54	(chart review or medical record review or (review adj5 patient records) or case series or consecutive patients or nonconsecutive patients).ti,ab.	274141
55	((Population-based or nationwide or community based or national or hospital based) adj3 (stud$3 or cohort$ or data or register$ or database$ or sample$)).ti,ab.	226296
56	(Database analys#s or database stud$ or claims data$).ti,ab.	18874
57	(single center or single centre or single institution or hospital$).ti.	352399
58	or/36-57	4157977
59	exp registries/	98348
60	(registries or registry or disease registr$3 or register$1).ti,ab,kf.	201122

61	or/59-60	237017
62	Health Surveys/	63083
63	exp population surveillance/	69979
64	exp "Surveys and Questionnaires"/	1050333
65	(health survey$1 or survey$1 or surveillance).ti,ab,kf.	787968
66	exp Seroepidemiologic Studies/	23739
67	Neonatal Screening/	10284
68	Mass screening/	104563
69	dried blood spot testing/	1516
70	((neonat$ or newborn$ or prenatal or maternal) adj3 screen$).ti,ab.	18222
71	((CMV or HCMV or CCMV or cytomegalovirus) adj3 screen$).ti,ab.	612
72	or/62-71	1611508
73	58 or 61 or 72	5116765
74	73 not (exp animals/ not humans/)	4926055
75	Mathematical Concepts/	4082
76	Models, Theoretical/	152296
77	exp Models, Statistical/	413503
78	Computer Simulation/	189775
79	model$.kw.	18917
80	(model or models or modelling or modeling).ti. not (((mouse or mice or murine or rat or rats or rabbit or rabbits or cat or cats or dog or dogs or swine or porcine or pig or pigs or piglet or piglets or lamb or lambs or cattle or bovine or monkey or monkeys or rhesus or macaque$ or nonhuman or "in vitro" or animal$ or organism$) adj3 model$).ti,kf. or exp Disease Models, Animal/)	419582

81	((mathematic$ or statistic$ or transmission$ or epidemiolog$ or epidemic$ or vaccination$ or immuni$ or theoretical$) adj3 model$).ti,ab,kf.	129315
82	((linear or nonlinear or static$ or dynamic$ or explicit or implicit or discrete$ or continuous$ or deterministic$ or probabilistic$ or stochastic$ or deductive or inductive or floating or strateg$ or nonstrateg$ or conceptual$ or quantitative$ or qualitative$ or individual$ or structured or catalytic) adj3 model$).ti,ab,kf.	205030
83	or/75-82	1213189
84	83 not (exp animals/ not humans/)	1087710
85	84 not (animals/ and (mouse or mice or murine or rat or rats or rabbit or rabbits or cat or cats or dog or dogs or swine or porcine or pig or pigs or piglet or piglets or lamb or lambs or cattle or bovine or monkey or monkeys or rhesus or macaque$ or nonhuman or "in vitro" or animal$ or organism$ or "in vivo").ti.)	1080980
86	Meta-Analysis as Topic/	18508
87	Systematic Review/	137586
88	Meta-Analysis/	121385
89	exp Technology Assessment, Biomedical/	11214
90	(meta analy$ or metaanaly$).tw.	184057
91	(systematic adj2 (review$1 or overview$1)).tw.	191540
92	exp Review Literature as Topic/	14690
93	(cochrane or embase or psychlit or psyclit or psychinfo or psycinfo or cinahl or cinhal or science citation index or bids or cancerlit or biosis or lilacs or web of science or scopus).ab.	176872
94	(reference list$ or bibliograph$ or hand-search$ or relevant journals or manual search$).ab.	44761
95	(selection criteria or eligibility criteria or data extraction).ab. and review/	33516

96	(((systematic$ or methodologic or quantitative or integrative or collaborative) adj5 (review or overview)) or technology assessment or bibliographic study).ti.	143743
97	or/86-96	409520
98	97 not (comment/ or letter/ or editorial/)	393331
99	98 not (exp animals/ not humans.sh.)	388736
100	exp Australia/	146136
101	Australia$.ti,bt,ab,kw,jw.	244745
102	exp Japan/	136254
103	Japan$.ti,bt,ab,kw,jw.	515422
104	Israel/	29458
105	Israel$.ti,bt,ab,kw,jw.	43936
106	or/100-105	877504
107	exp Canada/	160249
108	Canada.ti,bt,ab,kw.	87879
109	Canadian$1.ti,bt,ab,jw,kf.	260409
110	(british columbia or alberta$ or saskatchewan or manitoba$ or ontario or quebec or new brunswick or nouveau brunswick or nova scotia or nouvelle ecosse or prince edward island or newfoundland or labrador or nunavut or nwt or northwest territories or yukon or nunavik or inuvialuit).ti,ab,kw,jw.	78728
111	(Abbotsford or Airdrie or Ajax or Aurora or Barrie or Belleville or Blainville or Brampton or Brantford or Brossard or Burlington or Burnaby or Caledon or Calgary or Cape Breton or Chatham Kent or Chilliwack or Clarington or Coquitlam or Drummondville or Edmonton or Fredericton or Fort McMurray or Gatineau or Granby or Grande Prairie or Sudbury or Guelph or Halton Hills or Iqaluit or Inuvik or Kamloops or Kawartha Lakes or Kelowna or Kingston or Kitchener or Langley	113930

	or Laval or Lethbridge or Levis or Longueuil or Maple Ridge or Markham or Medicine Hat or Milton or Mirabel or Mississauga or Moncton or Montreal or Nanaimo or New Westminster or Newmarket or Niagara Falls or Norfolk County or North Bay or North Vancouver or North Vancouver or Oakville or Oshawa or Ottawa or Peterborough or Pickering or Port Coquitlam or Prince George or Quebec City or Red Deer or Regina or Repentigny or Richmond or Richmond Hill or Saanich or Saguenay or Saint John or Saint-Hyacinthe or Saint-Jean-sur-Richelieu or Saint-Jerome or Sarnia or Saskatoon or Sault Ste Marie or Sherbrooke or St Albert or St Catharines or St John's or Strathcona County or Surrey or Terrebonne or Thunder Bay or Toronto or Trois-Rivieres or Vancouver or Vaughan or ((Cambridge or (Halifax or Hamilton or London or Victoria or Waterloo or Welland or Whitby or Windsor)) not (UK or Britain or United Kingdom or England or Australia)) or Whitehorse or Winnipeg or Wood Buffalo or Yellowknife).ti,ab,kw.	
112	(Abbotsford or Airdrie or Ajax or Aurora or Barrie or Belleville or Blainville or Brampton or Brantford or Brossard or Burlington or Burnaby or Caledon or Calgary or Cape Breton or Chatham Kent or Chilliwack or Clarington or Coquitlam or Drummondville or Edmonton or Fredericton or Fort McMurray or Gatineau or Granby or Grande Prairie or Sudbury or Guelph or Halton Hills or Iqaluit or Inuvik or Kamloops or Kawartha Lakes or Kelowna or Kingston or Kitchener or Langley or Laval or Lethbridge or Levis or Longueuil or Maple Ridge or Markham or Medicine Hat or Milton or Mirabel or Mississauga or Moncton or Montreal or Nanaimo or New Westminster or Newmarket or Niagara Falls or Norfolk County or North Bay or North Vancouver or North Vancouver or Oakville or Oshawa or Ottawa or Peterborough or Pickering or Port Coquitlam or Prince George or Quebec City or Red Deer or Regina or Repentigny or Richmond or Richmond Hill or Saanich or Saguenay or Saint John or Saint-Hyacinthe or Saint-Jean-sur-Richelieu or Saint-Jerome or Sarnia or Saskatoon or Sault Ste Marie or Sherbrooke or St Albert or St Catharines or St John's or Strathcona County or Surrey or Terrebonne or Thunder Bay or Toronto or Trois-Rivieres or Vancouver or Vaughan or ((Cambridge or (Halifax or Hamilton or London or Victoria or Waterloo or Welland or Whitby or Windsor)) not (UK or Britain or United Kingdom or England or Australia)) or Whitehorse or Winnipeg or Wood Buffalo or Yellowknife).ti,ab,kw.	113930
113	exp United States/	1358558

114	(united states or usa or us national or us census or us state$ or US cohort$ or US population or US adult$ or US children).ti,ab,kw.	381782
115	(US or (U adj S)).ti.	68472
116	(America$ not (Central America$ or South America$ or Latin America$)).ti.	109659
117	((midatlantic or mid-atlantic or middle-atlantic or Midwest$3 or northeast$3 or northwest$3 or southeast$3 or southern or southwest$3) adj (state$1 or region$ or us or usa)).ti,ab,kw.	15854
118	(Appalachia$ or great lakes or great plains or heartland or new England or pacific northwest or deep south or black belt or rust belt or district of Columbia or Washington DC or Alabama or Alaska or Arizona or Arkansas or little rock or California or san Francisco or San Diego or Los Angeles or Colorado or Connecticut or Florida or Hawaii or Honolulu or Idaho or Gainesville or Jacksonville or Tampa or Tallahassee or Georgia or Atlanta or Illinois or Chicago or Indiana or Indianapolis or West Lafayette or iowa or Kansas or Wichita or Kentucky or Louisiana or new Orleans or baton rouge or Shreveport or Maine or Orono or Johns Hopkins or Massachusetts or Boston or Harvard or Michigan or Detroit or Ann Arbor or East Lansing or Minnesota or Minneapolis or Rochester or Montana or Missisippi or Missouri or Missoula or Nebraska or Nevada or Las Vegas or New Hampshire or New Jersey or New Mexico or New York or North Carolina or North Dakota or Ohio or Cincinnati or Oklahoma or Oregon or Illinois or Chicago or Maryland or Pennsylvania or Philadelphia or South Carolina or South Dakota or Tennessee or Nashville or Memphis or Texas or Houston or Utah or Vermont or Virginia or Rhode Island or Washington or Seattle or West Virginia or Wisconsin or Wyoming or Delaware or Mayo Clinic or AHRQ).ti,ab,kw.	508651
119	(((Birmingham or Montgomery) adj al) or Huntsville or anchorage or fairmbanks or Phoenix or Tuscon or Flagstaff or Berkeley or Stanford or Vail or Denver or Farmington or New Haven or Hartford or Wilmington or Newark or Miami or ((Athens or Augusta) adj ga) or Boise or Urbana or Evanston or Lexington or Louisville or Bardstown or (Scarborough adj me) or Bethesda or Baltimore or Rockville or (Worcester adj ma) or Burlington or St Paul or Saint Paul or (Jackson adj	91648

	ms) or (Columbia adj mo) or Bozeman or Omaha or Lincoln or Columbus or Cleveland or Portland or Hershey or providence or Richmond or Washington).ti,ab,kw.	
120	Dollar$.ti,ab.	20319
121	(medicare or medicaid).ti,ab.	60588
122	North America/	20434
123	or/107-122	2381156
124	exp Europe/	1428598
125	European Union/	16303
126	(Europe$3 or EU5).ti,ab,bt,kf,jw.	850807
127	exp France/	101521
128	(France or french).ti,bt,ab,kw,jw.	141553
129	exp Germany/	119708
130	German$1.ti,bt,ab,kf,jw.	142372
131	exp Italy/	97093
132	(Italy or Italian$).ti,bt,ab,kf,jw.	182122
133	exp Spain/	77699
134	(Spain or Spanish or Spaniards).ti,bt,ab,kw,jw.	107844
135	exp United Kingdom/	367536
136	(UK or U K).ti.	30380
137	(Britain$ or (British$ not "British Columbia") or United Kingdom$ or (England$ not "New England") or Northern Ireland$ or Northern Irish$ or Scotland$ or Scottish$ or ((Wales or "South Wales") not "New South Wales") or Welsh$).ti,ab,kw,jw.	681750
138	(English adj3 (Society or Association or patients)).ti,ab.	2507

139	(English adj (population or hospital$1 or citizen$)).ti,ab.	700
140	"National Institute for Health and Care Excellence".ti,ab.	1956
141	(National Health Service$ or nhs$).ti,ab.	43472
142	(((Albania$ or Andorra$ or Armenia$ or Austria$ or Azerbaijan$ or Belarus$ or Belgium or Belgian$ or Bosnia$) and Herzegovin$) or Bulgaria$ or Croatia$ or Cyprus or Cyprian$ or Czechia$ or Denmark or Danish or Estonia$ or Finland or Finnish or Georgia$ or Greece or Greek$ or Hungary or Hungarian$ or Iceland$ or Ireland or Irish or Kosovo$ or Latvia$ or Liechtenstein or Lithuania$ or Luxembourg$ or Malta or Moldova$ or Monaco or Montenegro or Netherlands or Dutch$ or North Macedonia$ or Norway or Norwegian$ or Poland or Polish or Portugal or Portuguese or Romania$ or San Marino or Serbia$ or Slovakia$ or Slovenia$ or Sweden or Swedish or Switzerland or Swiss or Turkey or Turkish or Ukrain$).ti,ab,kf,jw.	627187
143	(euro or euros or EUR).ti,ab.	18001
144	or/124-143	3274631
145	exp South America/	164209
146	exp Central America/	15845
147	Carribean Region/ or West Indies/ or Dominican Republic/	5086
148	Latin America/	11260
149	(Latin? America$ or Latinoamerica$ or South America$ or Central America$ or Caribbean$).ti,bt,ab,kw,jw.	75679
150	(Argentina or Argentinian$ or Bolivia$ or Brazil$ or Brasil$ or Chile or Chilean$ or Colombia$ or Ecuador or Ecuadorian$ or Paraguay$ or Peru or Peruvian$ or Urugay$ or Venezuela$ or Costa Rica$ or El Salvador or Salvadorian$ or Guatemala$ or Guatemaltec$ or Hondura$ or Mexico or Mexican$ or Nicaragua$ or Panama$ or Dominican Republic or Dominican$).ti,bt,ab,kw,jw.	405238

151	(Buenos Aires or La Paz or Sucre or Rio de Janeiro or Sao Paulo or Santiago or Valparaiso or Concepcion or Bogota or Quito or Asuncion or Lima or Montevideo or Caracas or San Salvador or Tegucigalpa or Managua or Santo Domingo).ti,bt,ab.	38093
152	or/145-151	499746
153	(Global$2 or worldwide or world wide).ti,ab,bt,kf.	643122
154	International.ti,bt.	74849
155	or/153-154	711714
156	106 or 123 or 144 or 152 or 155	7131150
157	74 or 85	5641997
158	156 and 157	2153283
159	99 or 158	2488216
160	10 and 35	12232
161	159 and 160	1956
162	limit 161 to yr="2000 -Current"	1537
163	limit 162 to English language	1445

Supplemental Table 2. Initial search strategy for Embase

	Embase
**Search Platform:**	Ovid
**Date of Search:**	October 27, 2020<br>[Last Database Update: October 26, 2020]
**Date Range Searched:**	1974 to current, restricted to 2000 to current
**Search Filters:**	In development of the search strategies the following search filters were used and partly modified:

	Systematic Reviews<br>- SIGN Search Strategy Systematic Reviews, OVID format. Scottish Intermediate Guidelines Network (SIGN), Filter Systematic Reviews, Available from: http://sign.ac.uk/search-filters.html (Word document last modified April 25, 2017; cited May 13, 2020) [slightly modified, supplemented with additional search terms]<br>Country restrictions:<br>- Campbell, Sandy. Filter to Retrieve Studies Related to Canada, Canadian Provinces, and the One Hundred Largest Canadian Centres from the OVID MEDLINE Database. John W. Scott Health Sciences Library, University of Alberta. Rev. March 06, 2020. Available from: http://guides.library.ualberta.ca/health-sciences-search-filters/geographic-filters [used, partly modified/adapted]<br>- UAB Libraries. PubMed via LHL: Hedges: Search filter United States (work in progress!) [Internet], Cited: October 19, 2020. Available from: https://guides.library.uab.edu/pubmed/hedges [used, partly modified/adapted]<br>- Ayiku L, Levay P, Hudson T, Craven J, Barrett E, Finnegan A and Adams R. The MEDLINE UK filter: development and validation of a geographic search filter to retrieve research about the UK from OVID MEDLINE. Health Information and Libraries Journal, 2017 34 (3): 200-216. [consulted, partly used/adapted]

#	Search Terms	Hits
1	*Cytomegalovirus/	12257
2	exp *Human Cytomegalovirus/	4110
3	exp *Cytomegalovirus Infection/	15640
4	*Cytomegalovirus antibody/	592

5	*congenital cytomegalovirus infection/	80
6	Cytomegalovirus/ and (Cytomegalovir$ or cmv or hcmv or ccmv or human herpesvirus 5 or human herpes virus 5 or human herpesvirus type 5 or human herpes virus type 5 or human betaherpesvirus 5 or human beta-herpesvirus 5 or HHV 5 or HHV5 or salivary gland virus$ or cytomegalic).ab.	23862
7	exp Human Cytomegalovirus/ and (Cytomegalovir$ or cmv or hcmv or ccmv or human herpesvirus 5 or human herpes virus 5 or human herpesvirus type 5 or human herpes virus type 5 or human betaherpesvirus 5 or human beta-herpesvirus 5 or HHV 5 or HHV5 or salivary gland virus$ or cytomegalic).ab.	6173
8	exp Cytomegalovirus Infections/ and (Cytomegalovir$ or cmv or hcmv or ccmv or human herpesvirus 5 or human herpes virus 5 or human herpesvirus type 5 or human herpes virus type 5 or human betaherpesvirus 5 or human beta-herpesvirus 5 or HHV 5 or HHV5 or salivary gland virus$ or cytomegalic).ab.	22383
9	Cytomegalovirus antibody/ and (Cytomegalovir$ or cmv or hcmv or ccmv or human herpesvirus 5 or human herpes virus 5 or human herpesvirus type 5 or human herpes virus type 5 or human betaherpesvirus 5 or human beta-herpesvirus 5 or HHV 5 or HHV5 or salivary gland virus$ or cytomegalic).ab.	1490
10	congenital cytomegalovirus infection/ and (Cytomegalovir$ or cmv or hcmv or ccmv or human herpesvirus 5 or human herpes virus 5 or human herpesvirus type 5 or human herpes virus type 5 or human betaherpesvirus 5 or human beta-herpesvirus 5 or HHV 5 or HHV5 or salivary gland virus$ or cytomegalic).ab.	75
11	(Cytomegalovir$ or cmv or hcmv or ccmv or human herpesvirus 5 or human herpes virus 5 or human herpesvirus type 5 or human herpes virus type 5 or human betaherpesvirus 5 or human beta-herpesvirus 5 or HHV 5 or HHV5 or salivary gland virus$).ti.	32775
12	Cytomegalovir$.ab. /freq=2	5799
13	Cytomegalovir$.af. and (cmv or hcmv or ccmv or hhv 5 or hhv5).ab. /freq=2	28246

14	(Cytomegalovir$ or cmv or hcmv or ccmv or human herpesvirus 5 or human herpes virus 5 or human herpesvirus type 5 or human herpes virus type 5 or human betaherpesvirus 5 or human beta-herpesvirus 5 or HHV 5 or HHV5 or salivary gland virus$).ab. and (article-in-press or conference abstract or in-process).st.	19306
15	or/1-14	63922
16	Cytomegalovirus infection/ep	1257
17	congenital cytomegalovirus infection/ep	12
18	Seroepidemiology/	4236
19	Congenital infection/ep	194
20	Congenital disorder/ep	575
21	Intrauterine infection/ep	167
22	Seroprevalence/	22356
23	Prevalence/ or Incidence/	1109154
24	mortality/ or childhood mortality/ or embryo mortality/ or fetus mortality/ or hospital mortality/ or infant mortality/ or mortality rate/ or perinatal mortality/ or premature mortality/ or prenatal mortality/ or standardized mortality ratio/	892003
25	Cytomegalovirus seropositivity/	1
26	Infection rate/	31638
27	Newborn Screening/ or Prenatal Screening/	27856
28	dried blood spot testing/	4053
29	Virus Shedding/	6939
30	(prevalence$ or prevalent or incidence$ or incident or mortalit$ or epidemiolog$ or morbidity or seroepidemiolog$ or seroprevalen$ or seroinciden$ or serosurvey$ or sero survey or serosurveillance or sero surveillance).ti,ab,kw.	3578999

31	(cases adj4 (birth$ or year$ or positive or symptomatic or asymptomatic)).ti,ab.	148133
32	(CMV cases or HCMV cases or CCMV cases).ti,ab.	86
33	((transmission or diagnosis) adj4 rate$).ti,ab.	24584
34	(transmission adj3 risk).mp.	16413
35	(infection rate$ or disease rate$).ti,ab,kw.	35995
36	Frequency.ti,ab.	1084455
37	((neonat$ or newborn$ or prenatal or maternal) adj2 screening).ti,ab.	23620
38	Shedding.ti,ab,kw.	30885
39	or/16-38	5041452
40	cohort analysis/	631612
41	exp longitudinal study/	147096
42	observational study/	211927
43	retrospective study/	984448
44	exp "medical record review"/	131597
45	prospective study/	638840
46	cross-sectional study/	375902
47	exp case control study/	181147
48	Factual database/	24636
49	Major clinical study/	3917630
50	Controlled study/	7775184
51	(Long-term or observational or cohort? or longitudinal or prospective or retrospective or comparative or case-control or cross-sectional or followup or survey$).ti.	1202651

52	(real-world or real life).ti.	30845
53	(cohort analys$ or cohort stud$ or longitudinal stud$ or chart review$ or medical record review$ or observational stud$ or retrospective analys$ or retrospective chart review? or retrospective clinical stud$ or retrospective cohort$ or retrospective stud$ or retrospective database$ or retrospective observational$ or cross-sectional stud$ or cross-sectional setting$ or case control stud$ or case control setting or nested case control stud$ or matched case-control stud$ or case series).kw.	48595
54	(cohort adj (study or studies or analy$)).ti,ab.	327648
55	(cohort$ adj2 (birth$ or newborn$)).ti,ab,kw.	23932
56	((longitudinal or cross sectional or case-control or pragmatic or large scale) adj (study or studies)).ti,ab.	471566
57	((observational or retrospective or prospective) adj3 (study or studies or cohort$1 or data$ or analys#s)).ti,ab.	1373187
58	((real world or real life) adj3 (data$ or setting or cohort$ or stud$ or experience$ or result$)).ti,ab.	45411
59	(data adj (report$ or data system$1)).ti,ab.	16089
60	Electronic Medical Record/	57246
61	(chart review or medical record review or (review adj5 patient records) or case series or consecutive patients or nonconsecutive patients).ti,ab,kw.	441587
62	((Population-based or nationwide or community based or national or hospital based) adj3 (stud$3 or cohort$ or data$ or register$ or database$ or sample$)).ti,ab.	321346
63	(Database analys#s or database stud$ or claims data$).ti,ab.	34416
64	(single center or single centre or single institution or hospital$).ti.	452825
65	or/40-64	11926188

66	Disease registry/	15169
67	register/	114486
68	(registries or registry or disease regist$3 or register$1 or national regist$).ti,ab,kw.	312980
69	or/66-68	331704
70	exp Health survey/	226423
71	exp disease surveillance/	30534
72	Population research/	110304
73	Newborn screening/	19436
74	dried blood spot testing/	4053
75	(health survey$1 or survey$1 or surveillance).ti,ab,hw.	1695350
76	Mass screening/ or Screening/	234002
77	Newborn screening/	19436
78	Prenatal screening/	8662
79	((neonat$ or newborn$ or prenatal or maternal) adj3 screen$).ti,ab.	27075
80	((CMV or HCMV or CCMV or cytomegalovirus) adj3 screen$).ti,ab.	913
81	Seroepidemiology/	4236
82	or/70-81	2056353
83	65 or 69 or 82	13205442
84	(exp Animal/ or animal experiment/ or nonhuman/) not (exp Human/ or Human experiment/)	6586246
85	animal experiment/ and (mouse or mice or murine or rat or rats or rabbit or rabbits or cat or cats or dog or dogs or swine or porcine or pig or pigs or piglet or piglets or lamb or lambs or cattle or bovine or monkey or monkeys).ti.	1089113

86	or/84-85	6638120
87	83 not 86	10453145
88	exp Mathematical model/	412331
89	Simulation/	186324
90	model$.kw.	283884
91	(model or models or modelling or modeling).ti. not (((mouse or mice or murine or rat or rats or rabbit or rabbits or cat or cats or dog or dogs or swine or porcine or pig or pigs or piglet or piglets or lamb or lambs or cattle or bovine or monkey or monkeys or rhesus or macaque$ or nonhuman or "in vitro" or animal$ or organism$) adj3 model$).ti,kw. or exp Animal Model/)	466020
92	((mathematic$ or statistic$ or transmission$ or epidemiolog$ or epidemic$ or vaccination$ or immuni$ or theoretical$) adj3 model$).ti,ab,kw.	151635
93	((linear or nonlinear or static$ or dynamic$ or explicit or implicit or discrete$ or continuous$ or deterministic$ or probabilistic$ or stochastic$ or deductive or inductive or floating or strateg$ or nonstrateg$ or conceptual$ or quantitative$ or qualitative$ or individual$ or structured or catalytic) adj3 model$).ti,ab,kw.	259436
94	or/88-93	1323804
95	94 not ((exp Animal/ or animal experiment/ or nonhuman/) not (exp Human/ or Human experiment/))	1098291
96	95 not (animal experiment/ and (mouse or mice or murine or rat or rats or rabbit or rabbits or cat or cats or dog or dogs or swine or porcine or pig or pigs or piglet or piglets or lamb or lambs or cattle or bovine or monkey or monkeys).ti.)	1094967
97	87 or 96	11084755
98	Systematic Review/	268566
99	exp Meta Analysis/	200567

100	biomedical technology assessment/	14706
101	((meta adj analy$) or metaanalys$).tw.	242391
102	(systematic adj2 (review$1 or overview$1)).tw.	241163
103	(cochrane or embase or psychlit or psyclit or psychinfo or psycinfo or cinahl or cinhal or science citation index or bids or cancerlit or biosis or lilacs or web of science or scopus).ab.	215318
104	(reference lists or bibliograph$ or hand search$ or manual search$ or relevant journals).ab.	53452
105	(selection criteria or eligibility criteria or data extraction).ab. and review.pt.	34229
106	(((systematic$ or methodologic or quantitative or integrative or collaborative) adj5 (review or overview)) or technology assessment or bibliographic study).ti.	174285
107	or/98-106	559338
108	(letter or editorial).pt.	1819537
109	(exp Animal/ or animal experiment/ or nonhuman/) not (exp Human/ or Human experiment/)	6586246
110	animal experiment/ and (mouse or mice or murine or rat or rats or rabbit or rabbits or cat or cats or dog or dogs or swine or porcine or pig or pigs or piglet or piglets or lamb or lambs or cattle or bovine or monkey or monkeys).ti.	1089113
111	or/108-110	8391193
112	107 not 111	537607
113	exp Australia/ or Australian/	177626
114	Australia$.ti,ab,kw,jw.	303508
115	exp Japan/	173060
116	"japanese (people)"/ or "Japanese (people)"/	29532
117	Japan$.ti,ab,kw,jw.	666680
118	Israel/ or Israeli/	32101

119	Israel$.ti,ab,kw,jw.	48717
120	or/113-119	1096406
121	exp Canada/	185135
122	Canada.ti,ab,kw.	115238
123	Canadian$1.ti,ab,jw.	240802
124	(CADTH or pCODR or INESSS).ti,ab.	435
125	(british columbia or alberta$ or saskatchewan or manitoba$ or ontario or quebec or new brunswick or nouveau brunswick or nova scotia or nouvelle ecosse or prince edward island or newfoundland or labrador or nunavut or nwt or northwest territories or yukon or nunavik or inuvialuit).ti,ab,kw,jw.	96552
126	(Abbotsford or Airdrie or Ajax or Aurora or Barrie or Belleville or Blainville or Brampton or Brantford or Brossard or Burlington or Burnaby or Caledon or Calgary or Cape Breton or Chatham Kent or Chilliwack or Clarington or Coquitlam or Drummondville or Edmonton or Fredericton or Fort McMurray or Gatineau or Granby or Grande Prairie or Sudbury or Guelph or Halton Hills or Iqaluit or Inuvik or Kamloops or Kawartha Lakes or Kelowna or Kingston or Kitchener or Langley or Laval or Lethbridge or Levis or Longueuil or Maple Ridge or Markham or Medicine Hat or Milton or Mirabel or Mississauga or Moncton or Montreal or Nanaimo or New Westminster or Newmarket or Niagara Falls or Norfolk County or North Bay or North Vancouver or North Vancouver or Oakville or Oshawa or Ottawa or Peterborough or Pickering or Port Coquitlam or Prince George or Quebec City or Red Deer or Regina or Repentigny or Richmond or Richmond Hill or Saanich or Saguenay or Saint John or Saint-Hyacinthe or Saint-Jean-sur-Richelieu or Saint-Jerome or Sarnia or Saskatoon or Sault Ste Marie or Sherbrooke or St Albert or St Catharines or St John's or Strathcona County or Surrey or Terrebonne or Thunder Bay or Toronto or Trois-Rivieres or Vancouver or Vaughan or ((Cambridge or (Halifax or Hamilton or London or Victoria or Waterloo or Welland or Whitby or Windsor)) not (UK or Britain or United Kingdom or England or Australia)) or Whitehorse or Winnipeg or Wood Buffalo or Yellowknife).ti,ab,kw.	174701

127	exp United States/	1233547
128	(united states or usa or us national or us census or us state$ or US cohort$ or US population or US adult$ or US children).ti,ab,kw.	548740
129	(US or (U adj S)).ti.	85366
130	(America$ not (Central America$ or South America$ or Latin America$)).ti.	125454
131	((midatlantic or mid-atlantic or middle-atlantic or Midwest$3 or northeast$3 or northwest$3 or southeast$3 or southern or southwest$3) adj (state$1 or region$ or us or usa)).ti,ab,kw.	19746
132	(Appalachia$ or great lakes or great plains or heartland or new England or pacific northwest or deep south or black belt or rust belt or district of Columbia or Washington DC or Alabama or Alaska or Arizona or Arkansas or little rock or California or san Francisco or San Diego or Los Angeles or Colorado or Connecticut or Florida or Hawaii or Honolulu or Idaho or Gainesville or Jacksonville or Tampa or Tallahassee or Georgia or Atlanta or Illinois or Chicago or Indiana or Indianapolis or West Lafayette or iowa or Kansas or Wichita or Kentucky or Louisiana or new Orleans or baton rouge or Shreveport or Maine or Orono or Johns Hopkins or Massachusetts or Boston or Harvard or Michigan or Detroit or Ann Arbor or East Lansing or Minnesota or Minneapolis or Rochester or Montana or Missisippi or Missouri or Missoula or Nebraska or Nevada or Las Vegas or New Hampshire or New Jersey or New Mexico or New York or North Carolina or North Dakota or Ohio or Cincinnati or Oklahoma or Oregon or Illinois or Chicago or Maryland or Pennsylvania or Philadelphia or South Carolina or South Dakota or Tennessee or Nashville or Memphis or Texas or Houston or Utah or Vermont or Virginia or Rhode Island or Washington or Seattle or West Virginia or Wisconsin or Wyoming or Delaware or Mayo Clinic or AHRQ).ti,ab,kw.	686699
133	(((Birmingham or Montgomery) adj al) or Huntsville or anchorage or fairmbanks or Phoenix or Tuscon or Flagstaff or Berkeley or Stanford or Vail or Denver or Farmington or New Haven or Hartford or Wilmington or Newark or Miami or ((Athens or Augusta) adj ga) or Boise or Urbana or Evanston or Lexington or Louisville or Bardstown or (Scarborough adj me) or Bethesda or Baltimore or Rockville or (Worcester adj ma) or Burlington or St Paul or Saint Paul or (Jackson adj	122149

	ms) or (Columbia adj mo) or Bozeman or Omaha or Lincoln or Columbus or Cleveland or Portland or Hershey or providence or Richmond or Washington).ti,ab,kw.	
134	Dollar$.ti,ab.	23287
135	(medicare or medicaid).ti,ab.	85169
136	North America/	35604
137	North America$.ti,ab,kw.	73512
138	exp North American/	167865
139	or/121-138	2765646
140	exp Europe/	1589093
141	exp European/ or exp EU Citizen/	180912
142	(Europe$3 or EU5).ti,ab,kw,jw.	1402876
143	exp France/ or Frenchman/	123535
144	France.ti,ab,kw.	95119
145	french.ti,ab,jw.	94639
146	exp Germany/ or "german (citizen)"/	187869
147	German$1.ti,ab,kw,jw.	248085
148	exp Italy/ or "italian (citizen)"/	118621
149	(Italy or Italian$).ti,ab,kw,jw.	234377
150	exp Spain/ or Spaniard/	99716
151	(Spain or Spanish or Spaniard$).ti,bt,ab,kw,jw.	139042
152	exp United Kingdom/ or exp British Citizen/	423515
153	(UK or U K).ti.	45264

154	(britain$ or (british$ not "british columbia") or united kingdom$ or (england$ not "new england") or northern ireland$ or northern irish$ or scotland$ or scottish$ or ((wales or "south wales") not "new south wales") or welsh$).ti,ab,kw,jw.	773210
155	(English adj3 (Society or Association or patients)).ti,ab.	3233
156	(English adj (population or hospital$1 or citizen$)).ti,ab.	928
157	"National Institute for Health and Care Excellence".ti,ab.	3435
158	(((Albania$ or Andorra$ or Armenia$ or Austria$ or Azerbaijan$ or Belarus$ or Belgium or Belgian$ or Bosnia$) and Herzegovin$) or Bulgaria$ or Croatia$ or Cyprus or Cyprian$ or Czechia$ or Denmark or Danish or Estonia$ or Finland or Finnish or Georgia$ or Greece or Greek$ or Hungary or Hungarian$ or Iceland$ or Ireland or Irish or Kosovo$ or Latvia$ or Liechtenstein or Lithuania$ or Luxembourg$ or Malta or Moldova$ or Monaco or Montenegro or Netherlands or Dutch$ or North Macedonia$ or Norway or Norwegian$ or Poland or Polish or Portugal or Portuguese or Romania$ or San Marino or Scandinavia$ or Serbia$ or Slovakia$ or Slovak Republic or Slovenia$ or Sweden or Swedish or Switzerland or Swiss or Turkey or Turkish or Ukrain$).ti,ab,kw,jw.	1160585
159	(euro or euros or EUR).ti,ab.	25923
160	or/140-159	4458308
161	exp "South and Central America"/	239029
162	exp South American/ or exp Central American/	16648
163	(Latin? America$ or Latinoamerica$ or South America$ or Central America$ or Caribbean$).ti,ab,od,kw,jw.	102333
164	(Argentina or Argentinian$ or Bolivia$ or Brazil$ or Brasil$ or Chile or Chilean$ or Colombia$ or Ecuador or Ecuadorian$ or Paraguay$ or Peru or Peruvian$ or Urugay$ or Venezuela$ or Costa Rica$ or El Salvador or Salvadorian$ or Guatemala$ or Guatemaltec$ or Hondura$ or Mexico or Mexican$ or Nicaragua$ or Panama$5 or Dominican Republic or Dominican$).ti,ab,od,kw,jw.	493744

165	(Buenos Aires or La Paz or Sucre or Rio de Janeiro or Sao Paulo or Santiago or Valparaiso or Concepcion or Bogota or Quito or Asuncion or Lima or Montevideo or Caracas or San Salvador or Tegucigalpa or Managua or Santo Domingo).ti,ab,jw.	61183
166	or/161-165	581245
167	(Global$2 or worldwide or world wide).ti,ab,hw.	866042
168	International.ti.	97848
169	or/167-168	955232
170	120 or 139 or 160 or 166 or 169	8943734
171	97 and 170	3588594
172	112 or 171	4037733
173	15 and 39	21231
174	172 and 173	4334
175	limit 174 to yr="2000 -Current"	3839
176	175 and conference abstract.pt,st.	1800
177	limit 176 to yr="2000 - 2016"	1150
178	175 not 177	2689
179	limit 178 to English language	2571

Supplemental Table 3. Initial search strategy for LILACS

	LILACS (Latin American and Caribbean Health Sciences Literature)<br><br>(Medline not included)	
**Search Platform:**	VHL – Virtual Library<br><br>https://lilacs.bvsalud.org/en/	
**Date of Search:**	October 27, 2020	
**Date Range Searched:**	2000-2020	
#	Search Terms	Hits
1	(mh:("Cytomegalovirus") OR mh:("Cytomegalovirus Infections") OR tw:("Cytomegalovirus") OR tw:("cmv") OR tw:("hcmv") OR tw:("ccmv") OR tw:("citomegalovirus") OR tw:("human herpesvirus 5") OR tw:("human herpes virus 5") OR tw:("human herpesvirus type 5") OR tw:("human herpes virus type 5") OR tw:("HHV 5") OR tw:("HHV5") OR tw:("salivary gland virus")) AND (mh:("Cytomegalovirus Infections/EP") OR mh:("Cytomegalovirus Infections/MO") OR mh:("Virus Diseases/EP") OR mh:("Virus Diseases/MO") OR mh:("Infant, Newborn, Diseases/EP") OR mh:("Infant, Newborn, Diseases/MO") OR mh:("Infant, Premature, Diseases/EP") OR mh:("Infant, Premature, Diseases/MO") OR mh:("Disease Transmission, Infectious/SN") OR mh:("Infectious Disease Transmission, Vertical/SN") OR mh:("Virus Shedding") OR mh:("Prevalence") OR mh:("Incidence") OR mh:("mortality") OR mh:("Seroepidemiologic Studies") OR mh:("Neonatal Screening") OR tw:(prevalenc*) OR tw:("prevalent") OR tw:(incidenc*) OR tw:("incident") OR tw:(mortalit*) OR tw:(epidemiolog*) OR tw:(seroepidemiolog*) OR tw:(seroprevalen*) OR tw:(seroinciden*) OR tw:(serosurvey*) OR tw:("sero-survey") OR tw:("serosurveillance") OR tw:("sero surveillance") OR tw:(seropositiv*) OR tw:("CMV cases") OR tw:("HCMV cases") OR tw:("CCMV cases") OR tw:(transmission rate*) OR tw:(infection rate*) OR tw:(disease rate*) OR tw:("frequency") OR tw:("neonatal screening") OR tw:("newborn screening") OR tw:("shedding") OR tw:("prenatal screening") OR tw:("maternal screening") OR tw:("dried blood spot testing")) AND (db:("LILACS" OR "SES-SP" OR "BRISA"))	522

2	(mh:("Cytomegalovirus") OR mh:("Cytomegalovirus Infections") OR tw:("Cytomegalovirus") OR tw:("cmv") OR tw:("hcmv") OR tw:("ccmv") OR tw:("citomegalovirus") OR tw:("human herpesvirus 5") OR tw:("human herpes virus 5") OR tw:("human herpesvirus type 5") OR tw:("human herpes virus type 5") OR tw:("HHV 5") OR tw:("HHV5") OR tw:("salivary gland virus")) AND (mh:("Cytomegalovirus Infections/EP") OR mh:("Cytomegalovirus Infections/MO") OR mh:("Virus Diseases/EP") OR mh:("Virus Diseases/MO") OR mh:("Infant, Newborn, Diseases/EP") OR mh:("Infant, Newborn, Diseases/MO") OR mh:("Infant, Premature, Diseases/EP") OR mh:("Infant, Premature, Diseases/MO") OR mh:("Disease Transmission, Infectious/SN") OR mh:("Infectious Disease Transmission, Vertical/SN") OR mh:("Virus Shedding") OR mh:("Prevalence") OR mh:("Incidence") OR mh:("mortality") OR mh:("Seroepidemiologic Studies") OR mh:("Neonatal Screening") OR tw:(prevalenc*) OR tw:("prevalent") OR tw:(incidenc*) OR tw:("incident") OR tw:(mortalit*) OR tw:(epidemiolog*) OR tw:(seroepidemiolog*) OR tw:(seroprevalen*) OR tw:(seroinciden*) OR tw:(serosurvey*) OR tw:("sero-survey") OR tw:("serosurveillance") OR tw:("sero surveillance") OR tw:(seropositiv*) OR tw:("CMV cases") OR tw:("HCMV cases") OR tw:("CCMV cases") OR tw:(transmission rate*) OR tw:(infection rate*) OR tw:(disease rate*) OR tw:("frequency") OR tw:("neonatal screening") OR tw:("newborn screening") OR tw:("shedding") OR tw:("prenatal screening") OR tw:("maternal screening") OR tw:("dried blood spot testing")) AND (db:("LILACS" OR "SES-SP" OR "BRISA")) AND (year_cluster:[2000 TO 2020])	356
3	(mh:("Cytomegalovirus") OR mh:("Cytomegalovirus Infections") OR tw:("Cytomegalovirus") OR tw:("cmv") OR tw:("hcmv") OR tw:("ccmv") OR tw:("citomegalovirus") OR tw:("human herpesvirus 5") OR tw:("human herpes virus 5") OR tw:("human herpesvirus type 5") OR tw:("human herpes virus type 5") OR tw:("HHV 5") OR tw:("HHV5") OR tw:("salivary gland virus")) AND (mh:("Cytomegalovirus Infections/EP") OR mh:("Cytomegalovirus Infections/MO") OR mh:("Virus Diseases/EP") OR mh:("Virus Diseases/MO") OR mh:("Infant, Newborn, Diseases/EP") OR mh:("Infant, Newborn, Diseases/MO") OR mh:("Infant, Premature, Diseases/EP") OR mh:("Infant, Premature, Diseases/MO") OR mh:("Disease Transmission, Infectious/SN") OR mh:("Infectious Disease Transmission, Vertical/SN") OR mh:("Virus Shedding") OR mh:("Prevalence") OR mh:("Incidence") OR mh:("mortality") OR mh:("Seroepidemiologic	108

	Studies") OR mh:("Neonatal Screening") OR tw:(prevalenc*) OR tw:("prevalent") OR tw:(incidenc*) OR tw:("incident") OR tw:(mortalit*) OR tw:(epidemiolog*) OR tw:(seroepidemiolog*) OR tw:(seroprevalen*) OR tw:(seroinciden*) OR tw:(serosurvey*) OR tw:("sero-survey") OR tw:("serosurveillance") OR tw:("sero surveillance") OR tw:(seropositiv*) OR tw:("CMV cases") OR tw:("HCMV cases") OR tw:("CCMV cases") OR tw:(transmission rate*) OR tw:(infection rate*) OR tw:(disease rate*) OR tw:("frequency") OR tw:("neonatal screening") OR tw:("newborn screening") OR tw:("shedding") OR tw:("prenatal screening") OR tw:("maternal screening") OR tw:("dried blood spot testing")) AND (db:("LILACS" OR "BRISA" OR "SES-SP") AND la:("en")) AND (year_cluster:[2000 TO 2020])	

Widened search strategies

Supplemental Table 4. Widened search strategy for Medline (includes Medline in Process, Pubmed Not Medline, In Data Review, Publisher)

	Ovid MEDLINE®
**Search Platform:**	Ovid
**Date of Search:**	December 14, 2020<br>[Last Database Update: December 11, 2020]
**Date Range Searched:**	1946 to current, restricted to 2000-2020
**Search Filters:**	In development of the search strategies the following search filters were used and partly modified:<br>Systematic Reviews<br>- SIGN Search Strategy Systematic Reviews, OVID format. Scottish Intermediate Guidelines Network (SIGN), Filter Systematic Reviews. Available from: http://sign.ac.uk/search-filters.html (Word document last

	modified April 25, 2017; cited May 13, 2020) [supplemented by additional search terms]<br>Country restrictions:<br>- Campbell, Sandy. Filter to Retrieve Studies Related to Canada, Canadian Provinces, and the One Hundred Largest Canadian Centres from the OVID MEDLINE Database. John W. Scott Health Sciences Library, University of Alberta. Rev. March 06, 2020. Available from: http://guides.library.ualberta.ca/health-sciences-search-filters/geographic-filters [used, partly modified]<br>- UAB Libraries. PubMed via LHL: Hedges: Search filter United States (work in progress!)[Internet], Cited: October 19, 2020. Available from: https://guides.library.uab.edu/pubmed/hedges [used, partly modified]<br>- Ayiku L, Levay P, Hudson T, Craven J, Barrett E, Finnegan A and Adams R. The MEDLINE UK filter: development and validation of a geographic search filter to retrieve research about the UK from OVID MEDLINE. Health Information and Libraries Journal, 2017 34 (3): 200-216. [consulted, partly used/adapted]

#	Search Terms	Hits
1	*Cytomegalovirus/	12980
2	exp *Cytomegalovirus Infections/	19943
3	Cytomegalovir$.kf.	3743
4	Cytomegalovirus/ and (Cytomegalovir$ or cmv or hcmv or ccmv or human herpesvirus 5 or human herpes virus 5 or human herpesvirus type 5 or human herpes virus type 5 or human betaherpesvirus 5 or human beta-herpesvirus 5 or HHV 5 or HHV5 or salivary gland virus$ or cytomegalic).ab.	16642
5	exp Cytomegalovirus Infections/ and (Cytomegalovir$ or cmv or hcmv or ccmv or human herpesvirus 5 or human herpes virus 5 or human herpesvirus type 5 or human herpes virus type 5	17708

	or human betaherpesvirus 5 or human beta-herpesvirus 5 or HHV 5 or HHV5 or salivary gland virus$ or cytomegalic).ab.	
6	(Cytomegalovir$ or cmv or hcmv or ccmv or human herpesvirus 5 or human herpes virus 5 or human herpesvirus type 5 or human herpes virus type 5 or human betaherpesvirus 5 or human beta-herpesvirus 5 or HHV 5 or HHV5 or salivary gland virus$).ti,kf.	27977
7	Cytomegalovir$.ab. /freq=2	4996
8	Cytomegalovir$.af. and (cmv or hcmv or ccmv or hhv 5 or hhv5).ab. /freq=2	20895
9	(Cytomegalovir$ or cmv or hcmv or ccmv or human herpesvirus 5 or human herpes virus 5 or human herpesvirus type 5 or human herpes virus type 5 or human betaherpesvirus 5 or human beta-herpesvirus 5 or HHV 5 or HHV5 or salivary gland virus$).ab. and ("in data review" or in process or publisher or "pubmed not medline").st.	3956
10	or/1-9	39889
11	exp Cytomegalovirus Infections/ep, mo	3375
12	Virus Diseases/ep, mo	4534
13	Infant, Newborn, Diseases/ep, mo	4540
14	Infant, Premature, Diseases/ep, mo	3666
15	Pregnancy Complications, Infectious/ep, mo, sn	7956
16	Infectious Disease Transmission, Vertical/sn	1159
17	Disease Transmission, Infectious/sn	778
18	exp prenatal diagnosis/sn	2039
19	Prevalence/	298610
20	Incidence/	267969
21	Mortality/ or Child Mortality/ or Fetal Mortality/ or Hospital Mortality.mp. or exp Infant Mortality/ or mortality, premature/ [mp=title, abstract, original title, name of substance word,	135092

	subject heading word, floating sub-heading word, keyword heading word, organism supplementary concept word, protocol supplementary concept word, rare disease supplementary concept word, unique identifier, synonyms]	
22	Seroepidemiologic Studies/	20911
23	Neonatal Screening/	10350
24	dried blood spot testing/	1540
25	Virus shedding/	3504
26	(prevalence$ or prevalent or incidence$ or incident or mortalit$ or epidemiolog$ or seroepidemiolog$ or seroprevalen$ or seroinciden$ or serosurvey$ or sero-survey$ or serosurveillance or sero surveillance or morbidity or seropositiv$).ti,ab,kf.	2613697
27	(cases adj4 (birth$ or year$ or positive or symptomatic or asymptomatic)).ti,ab.	94560
28	(CMV cases or HCMV cases or CCMV cases).ti,ab.	51
29	((transmission or diagnosis) adj4 rate$).ti,ab.	17458
30	(transmission adj3 risk).mp.	13192
31	(infection rate$ or disease rate$).ti,ab,kf.	27333
32	Frequency.ti,ab.	844420
33	((neonat$ or newborn$ or prenatal or maternal) adj2 screening).ti,ab.	16150
34	Shedding.ti,ab,kf.	26482
35	or/11-34	3591794
36	Epidemiologic studies/	8484
37	exp case control studies/	1123668
38	exp cohort studies/	2061902
39	Retrospective Studies/	855466

40	Cross-sectional studies/	345417
41	observational studies as topic/ or observational study/	94584
42	evaluation studies/	255132
43	Databases, Factual/ or Data Collection/	172209
44	(Long-term or observational or cohort? or longitudinal or prospective or retrospective or comparative or case-control or cross-sectional or survey$).ti.	949679
45	(real-world or real life).ti.	14811
46	(cohort analys$ or cohort stud$ or longitudinal stud$ or chart review$ or medical record review$ or observational stud$ or retrospective analys$ or retrospective chart review? or retrospective clinical stud$ or retrospective cohort$ or retrospective stud$ or retrospective database$ or retrospective observational$ or cross-sectional stud$ or cross-sectional setting$ or case control stud$ or case control setting or nested case control stud$ or matched case-control stud$).kf.	25801
47	(cohort adj (study or studies or analy$)).ti,ab.	228073
48	(cohort$ adj2 (birth$ or newborn$)).ti,ab,kw.	18883
49	((longitudinal or cross sectional or case-control or pragmatic or large scale) adj (study or studies)).ti,ab.	363700
50	((observational or retrospective or prospective) adj3 (study or studies or cohort$1 or data$ or analys#s)).ti,ab.	885570
51	((real world or real life) adj3 (data$ or setting or cohort$ or stud$ or experience$ or result$)).ti,ab.	22053
52	(data adj (report$ or data system$1)).ti,ab.	11651
53	Electronic health records/	20670
54	(chart review or medical record review or (review adj5 patient records) or case series or consecutive patients or nonconsecutive patients).ti,ab.	277731

55	((Population-based or nationwide or community based or national or hospital based) adj3 (stud$3 or cohort$ or data or register$ or database$ or sample$)).ti,ab.	230038
56	(Database analys#s or database stud$ or claims data$).ti,ab.	19291
57	(single center or single centre or single institution or hospital$).ti.	356867
58	or/36-57	4204061
59	exp registries/	99324
60	(registries or registry or disease registr$3 or register$1).ti,ab,kf.	204624
61	or/59-60	240800
62	Health Surveys/	63381
63	exp population surveillance/	70355
64	exp "Surveys and Questionnaires"/	1057916
65	(health survey$1 or survey$1 or surveillance).ti,ab,kf.	799674
66	exp Seroepidemiologic Studies/	23865
67	Neonatal Screening/	10350
68	Mass screening/	105122
69	dried blood spot testing/	1540
70	((neonat$ or newborn$ or prenatal or maternal) adj3 screen$).ti,ab.	18499
71	((CMV or HCMV or CCMV or cytomegalovirus) adj3 screen$).ti,ab.	617
72	or/62-71	1628559
73	58 or 61 or 72	5173205
74	73 not (exp animals/ not humans/)	4981629
75	Mathematical Concepts/	4104

76	Models, Theoretical/	152959
77	exp Models, Statistical/	415883
78	Computer Simulation/	190572
79	model$.kw.	19445
80	(model or models or modelling or modeling).ti. not (((mouse or mice or murine or rat or rats or rabbit or rabbits or cat or cats or dog or dogs or swine or porcine or pig or pigs or piglet or piglets or lamb or lambs or cattle or bovine or monkey or monkeys or rhesus or macaque$ or nonhuman or "in vitro" or animal$ or organism$) adj3 model$).ti,kf. or exp Disease Models, Animal/)	425294
81	((mathematic$ or statistic$ or transmission$ or epidemiolog$ or epidemic$ or vaccination$ or immuni$ or theoretical$) adj3 model$).ti,ab,kf.	131135
82	((linear or nonlinear or static$ or dynamic$ or explicit or implicit or discrete$ or continuous$ or deterministic$ or probabilistic$ or stochastic$ or deductive or inductive or floating or strateg$ or nonstrateg$ or conceptual$ or quantitative$ or qualitative$ or individual$ or structured or catalytic) adj3 model$).ti,ab,kf.	208783
83	or/75-82	1225858
84	83 not (exp animals/ not humans/)	1099786
85	84 not (animals/ and (mouse or mice or murine or rat or rats or rabbit or rabbits or cat or cats or dog or dogs or swine or porcine or pig or pigs or piglet or piglets or lamb or lambs or cattle or bovine or monkey or monkeys or rhesus or macaque$ or nonhuman or "in vitro" or animal$ or organism$ or "in vivo").ti.)	1093007
86	Meta-Analysis as Topic/	18668
87	Systematic Review/	140336
88	Meta-Analysis/	123148
89	exp Technology Assessment, Biomedical/	11228

90	(meta analy$ or metaanaly$).tw.	188708
91	(systematic adj2 (review$1 or overview$1)).tw.	197318
92	exp Review Literature as Topic/	14904
93	(cochrane or embase or psychlit or psyclit or psychinfo or psycinfo or cinahl or cinhal or science citation index or bids or cancerlit or biosis or lilacs or web of science or scopus).ab.	182044
94	(reference list$ or bibliograph$ or hand-search$ or relevant journals or manual search$).ab.	45378
95	(selection criteria or eligibility criteria or data extraction).ab. and review/	33837
96	(((systematic$ or methodologic or quantitative or integrative or collaborative) adj5 (review or overview)) or technology assessment or bibliographic study).ti.	148538
97	or/86-96	418639
98	97 not (comment/ or letter/ or editorial/)	402200
99	98 not (exp animals/ not humans.sh.)	397530
100	exp Australia/	147128
101	Australia$.ti,bt,ab,kw,jw.	246696
102	exp Japan/	137158
103	Japan$.ti,bt,ab,kw,jw.	518057
104	Israel/	29593
105	Israel$.ti,bt,ab,kw,jw.	44300
106	or/100-105	882734
107	exp Canada/	161041
108	Canada.ti,bt,ab,kw.	88995
109	Canadian$1.ti,bt,ab,jw,kf.	261725

110	(british columbia or alberta$ or saskatchewan or manitoba$ or ontario or quebec or new brunswick or nouveau brunswick or nova scotia or nouvelle ecosse or prince edward island or newfoundland or labrador or nunavut or nwt or northwest territories or yukon or nunavik or inuvialuit).ti,ab,kw,jw.	79587
111	(Abbotsford or Airdrie or Ajax or Aurora or Barrie or Belleville or Blainville or Brampton or Brantford or Brossard or Burlington or Burnaby or Caledon or Calgary or Cape Breton or Chatham Kent or Chilliwack or Clarington or Coquitlam or Drummondville or Edmonton or Fredericton or Fort McMurray or Gatineau or Granby or Grande Prairie or Sudbury or Guelph or Halton Hills or Iqaluit or Inuvik or Kamloops or Kawartha Lakes or Kelowna or Kingston or Kitchener or Langley or Laval or Lethbridge or Levis or Longueuil or Maple Ridge or Markham or Medicine Hat or Milton or Mirabel or Mississauga or Moncton or Montreal or Nanaimo or New Westminster or Newmarket or Niagara Falls or Norfolk County or North Bay or North Vancouver or North Vancouver or Oakville or Oshawa or Ottawa or Peterborough or Pickering or Port Coquitlam or Prince George or Quebec City or Red Deer or Regina or Repentigny or Richmond or Richmond Hill or Saanich or Saguenay or Saint John or Saint-Hyacinthe or Saint-Jean-sur-Richelieu or Saint-Jerome or Sarnia or Saskatoon or Sault Ste Marie or Sherbrooke or St Albert or St Catharines or St John's or Strathcona County or Surrey or Terrebonne or Thunder Bay or Toronto or Trois-Rivieres or Vancouver or Vaughan or ((Cambridge or (Halifax or Hamilton or London or Victoria or Waterloo or Welland or Whitby or Windsor)) not (UK or Britain or United Kingdom or England or Australia)) or Whitehorse or Winnipeg or Wood Buffalo or Yellowknife).ti,ab,kw.	115323
112	(Abbotsford or Airdrie or Ajax or Aurora or Barrie or Belleville or Blainville or Brampton or Brantford or Brossard or Burlington or Burnaby or Caledon or Calgary or Cape Breton or Chatham Kent or Chilliwack or Clarington or Coquitlam or Drummondville or Edmonton or Fredericton or Fort McMurray or Gatineau or Granby or Grande Prairie or Sudbury or Guelph or Halton Hills or Iqaluit or Inuvik or Kamloops or Kawartha Lakes or Kelowna or Kingston or Kitchener or Langley or Laval or Lethbridge or Levis or Longueuil or Maple Ridge or Markham or Medicine Hat or Milton or Mirabel or Mississauga or Moncton or Montreal or Nanaimo or New Westminster or Newmarket or Niagara Falls or Norfolk County or North Bay or North Vancouver or North	115323

	Vancouver or Oakville or Oshawa or Ottawa or Peterborough or Pickering or Port Coquitlam or Prince George or Quebec City or Red Deer or Regina or Repentigny or Richmond or Richmond Hill or Saanich or Saguenay or Saint John or Saint-Hyacinthe or Saint-Jean-sur-Richelieu or Saint-Jerome or Sarnia or Saskatoon or Sault Ste Marie or Sherbrooke or St Albert or St Catharines or St John's or Strathcona County or Surrey or Terrebonne or Thunder Bay or Toronto or Trois-Rivieres or Vancouver or Vaughan or ((Cambridge or (Halifax or Hamilton or London or Victoria or Waterloo or Welland or Whitby or Windsor)) not (UK or Britain or United Kingdom or England or Australia)) or Whitehorse or Winnipeg or Wood Buffalo or Yellowknife).ti,ab,kw.	
113	exp United States/	1363122
114	(united states or usa or us national or us census or us state$ or US cohort$ or US population or US adult$ or US children).ti,ab,kw.	386833
115	(US or (U adj S)).ti.	69485
116	(America$ not (Central America$ or South America$ or Latin America$)).ti.	110613
117	((midatlantic or mid-atlantic or middle-atlantic or Midwest$3 or northeast$3 or northwest$3 or southeast$3 or southern or southwest$3) adj (state$1 or region$ or us or usa)).ti,ab,kw.	16121
118	(Appalachia$ or great lakes or great plains or heartland or new England or pacific northwest or deep south or black belt or rust belt or district of Columbia or Washington DC or Alabama or Alaska or Arizona or Arkansas or little rock or California or san Francisco or San Diego or Los Angeles or Colorado or Connecticut or Florida or Hawaii or Honolulu or Idaho or Gainesville or Jacksonville or Tampa or Tallahassee or Georgia or Atlanta or Illinois or Chicago or Indiana or Indianapolis or West Lafayette or iowa or Kansas or Wichita or Kentucky or Louisiana or new Orleans or baton rouge or Shreveport or Maine or Orono or Johns Hopkins or Massachusetts or Boston or Harvard or Michigan or Detroit or Ann Arbor or East Lansing or Minnesota or Minneapolis or Rochester or Montana or Missisippi or Missouri or Missoula or Nebraska or Nevada or Las Vegas or New Hampshire or New Jersey or New Mexico or New York or North Carolina or North Dakota or Ohio or Cincinnati or Oklahoma or Oregon or Illinois or Chicago or Maryland or Pennsylvania or Philadelphia or South Carolina or South Dakota or Tennessee or	512919

	Nashville or Memphis or Texas or Houston or Utah or Vermont or Virginia or Rhode Island or Washington or Seattle or West Virginia or Wisconsin or Wyoming or Delaware or Mayo Clinic or AHRQ).ti,ab,kw.	
119	(((Birmingham or Montgomery) adj al) or Huntsville or anchorage or fairmbanks or Phoenix or Tuscon or Flagstaff or Berkeley or Stanford or Vail or Denver or Farmington or New Haven or Hartford or Wilmington or Newark or Miami or ((Athens or Augusta) adj ga) or Boise or Urbana or Evanston or Lexington or Louisville or Bardstown or (Scarborough adj me) or Bethesda or Baltimore or Rockville or (Worcester adj ma) or Burlington or St Paul or Saint Paul or (Jackson adj ms) or (Columbia adj mo) or Bozeman or Omaha or Lincoln or Columbus or Cleveland or Portland or Hershey or providence or Richmond or Washington).ti,ab,kw.	92457
120	Dollar$.ti,ab.	20506
121	(medicare or medicaid).ti,ab.	61209
122	North America/	20545
123	or/107-122	2397111
124	exp Europe/	1434994
125	European Union/	16351
126	(Europe$3 or EU5).ti,ab,bt,kf,jw.	859470
127	exp France/	101999
128	(France or french).ti,bt,ab,kw,jw.	142955
129	exp Germany/	120308
130	German$1.ti,bt,ab,kf,jw.	143978
131	exp Italy/	97856
132	(Italy or Italian$).ti,bt,ab,kf,jw.	183823
133	exp Spain/	78284

134	(Spain or Spanish or Spaniards).ti,bt,ab,kw,jw.	109307
135	exp United Kingdom/	368611
136	(UK or U K).ti.	30880
137	(Britain$ or (British$ not "British Columbia") or United Kingdom$ or (England$ not "New England") or Northern Ireland$ or Northern Irish$ or Scotland$ or Scottish$ or ((Wales or "South Wales") not "New South Wales") or Welsh$).ti,ab,kw,jw.	684812
138	(English adj3 (Society or Association or patients)).ti,ab.	2532
139	(English adj (population or hospital$1 or citizen$)).ti,ab.	713
140	"National Institute for Health and Care Excellence".ti,ab.	2004
141	(National Health Service$ or nhs$).ti,ab.	43958
142	(((Albania$ or Andorra$ or Armenia$ or Austria$ or Azerbaijan$ or Belarus$ or Belgium or Belgian$ or Bosnia$) and Herzegovin$) or Bulgaria$ or Croatia$ or Cyprus or Cyprian$ or Czechia$ or Denmark or Danish or Estonia$ or Finland or Finnish or Georgia$ or Greece or Greek$ or Hungary or Hungarian$ or Iceland$ or Ireland or Irish or Kosovo$ or Latvia$ or Liechtenstein or Lithuania$ or Luxembourg$ or Malta or Moldova$ or Monaco or Montenegro or Netherlands or Dutch$ or North Macedonia$ or Norway or Norwegian$ or Poland or Polish or Portugal or Portuguese or Romania$ or San Marino or Serbia$ or Slovakia$ or Slovenia$ or Sweden or Swedish or Switzerland or Swiss or Turkey or Turkish or Ukrain$).ti,ab,kf,jw.	633755
143	(euro or euros or EUR).ti,ab.	18177
144	or/124-143	3298564
145	exp South America/	165578
146	exp Central America/	15908
147	Carribean Region/ or West Indies/ or Dominican Republic/	5119
148	Latin America/	11334

149	(Latin? America$ or Latinoamerica$ or South America$ or Central America$ or Caribbean$).ti,bt,ab,kw,jw.	76482
150	(Argentina or Argentinian$ or Bolivia$ or Brazil$ or Brasil$ or Chile or Chilean$ or Colombia$ or Ecuador or Ecuadorian$ or Paraguay$ or Peru or Peruvian$ or Urugay$ or Venezuela$ or Costa Rica$ or El Salvador or Salvadorian$ or Guatemala$ or Guatemaltec$ or Hondura$ or Mexico or Mexican$ or Nicaragua$ or Panama$ or Dominican Republic or Dominican$).ti,bt,ab,kw,jw.	410136
151	(Buenos Aires or La Paz or Sucre or Rio de Janeiro or Sao Paulo or Santiago or Valparaiso or Concepcion or Bogota or Quito or Asuncion or Lima or Montevideo or Caracas or San Salvador or Tegucigalpa or Managua or Santo Domingo).ti,bt,ab.	38618
152	or/145-151	505355
153	(Global$2 or worldwide or world wide).ti,ab,bt,kf.	657621
154	International.ti,bt.	75585
155	or/153-154	726832
156	106 or 123 or 144 or 152 or 155	7189688
157	74 or 85	5705803
158	156 and 157	2177379
159	99 or 158	2519970
160	10 and 35	12353
161	159 and 160	1975
162	limit 161 to yr="2000 -Current"	1556
163	limit 162 to English language	1463
164	((neonat$ or newborn$ or prenatal or antenatal or postnatal or maternal) adj3 screen$).ti,ab.	20484
165	(reactivat$ or re-activat$).ti,ab,kf.	44290

166	Force of infection$.ti,ab,kf.	531
167	(Pregnancy loss$ or miscarriage$ or abortion$ or f?etal death$).ti,ab,kf.	87259
168	((targeted or universal or routine) adj2 screen$).ti,ab.	18325
169	(sequelae or long term effects).ti,ab,kf.	95069
170	((emotional$ or psychologic$ or psychosocial$ or social or psychometric or neuropsycholog$ or neurodevelopment$ or development$ or neurocognit$ or cognit$ or mental or language or auditory or audiolog$ or hearing) adj3 (outcome$ or function$ or performance or skills)).ti,ab.	276811
171	(cognitive abilit$ or cognitive disabilit$ or cognitive delay$ or mental development or developmental disabilit$ or developmental abnormalit$ or audiologic?? outcome$ or hearing deficits or hearing impairment or hearing loss or hearing abilit$ or phonologic?? outcome$ or auditory perception or speech recognition or word recognition or speech discrimination or language production or language delay or speech perception or language perception or language development or (language adj2 processing) or speech Intelligibility or speech development or verbal ability or (child adj2 development) or emotional problems or peer problems or executive function$ or working memory or cognitive flexibility or flexible thinking or psychological development or mental health or school performance).ti,ab.	323738
172	(Polymerase Chain Reaction/ or Real-Time Polymerase Chain Reaction/ or (Polymerase Chain Reaction or PCR or test$ or screen$).ti,ab,kf.) and (urin$ or saliva$ or oral swab$ or blood spot$).ti,ab.	131399
173	Alethia$.ti,ab,kf.	4
174	or/164-173	920624
175	35 or 174	4302503
176	(Polymerase Chain Reaction/ or Real-Time Polymerase Chain Reaction/ or (Polymerase Chain Reaction or PCR or test$ or screen$).ti,ab,kf.) and (urin$ or saliva$ or oral swab$ or blood spot$).ti,ab.	131399

177	Alethia$.ti,ab,kf.	4
178	((neonat$ or newborn$ or prenatal or antenatal or postnatal or maternal) adj3 screen$).ti,ab.	20484
179	((targeted or universal or routine) adj2 screen$).ti,ab.	18325
180	or/176-179	165409
181	58 or 61 or 72 or 85 or 180	5989412
182	181 not (exp animals/ not humans.sh.)	5782405
183	156 and 182	2194090
184	99 or 183	2536297
185	10 and 175	15004
186	184 and 185	2244
187	limit 186 to yr="2000 -Current"	1762
188	limit 187 to English language	1654

Supplemental Table 5. Widened search strategy for Embase

	Embase
**Search Platform:**	Ovid
**Date of Search:**	December 14, 2020<br><br>[Last Database Update: December 11, 2020]
**Date Range Searched:**	1974 to current, restricted to 2000 to current
**Search Filters:**	In development of the search strategies the following search filters were used and partly modified:<br><br>Systematic Reviews<br><br>- SIGN Search Strategy Systematic Reviews, OVID format. Scottish Intermediate Guidelines Network (SIGN), Filter Systematic Reviews. Available from: http://sign.ac.uk/search-filters.html [Word document last modified April 25, 2017; cited May 13, 2020) [slightly modified, supplemented with additional search terms]<br><br>Country restrictions:<br><br>- Campbell, Sandy. Filter to Retrieve Studies Related to Canada, Canadian Provinces, and the One Hundred Largest Canadian Centres from the OVID MEDLINE Database. John W. Scott Health Sciences Library, University of Alberta. Rev. March 06, 2020. Available from: http://guides.library.ualberta.ca/health-sciences-search-filters/geographic-filters [used, partly modified/adapted]<br>- UAB Libraries. PubMed via LHL: Hedges: Search filter United States (work in progress!) [Internet], Cited: October 19, 2020. Available from: https://guides.library.uab.edu/pubmed/hedges [used, partly modified/adapted]<br>- Ayiku L, Levay P, Hudson T, Craven J, Barrett E, Finnegan A and Adams R. The MEDLINE UK filter: development and validation of a geographic search

	filter to retrieve research about the UK from OVID MEDLINE. Health Information and Libraries Journal, 2017 34 (3): 200-216. [consulted, partly used/adapted]

#	Search Terms	Hits
1	*Cytomegalovirus/	12403
2	exp *Human Cytomegalovirus/	4129
3	exp *Cytomegalovirus Infection/	15669
4	*Cytomegalovirus antibody/	595
5	*congenital cytomegalovirus infection/	82
6	Cytomegalovirus/ and (Cytomegalovir$ or cmv or hcmv or ccmv or human herpesvirus 5 or human herpes virus 5 or human herpesvirus type 5 or human herpes virus type 5 or human betaherpesvirus 5 or human beta-herpesvirus 5 or HHV 5 or HHV5 or salivary gland virus$ or cytomegalic).ab.	24047
7	exp Human Cytomegalovirus/ and (Cytomegalovir$ or cmv or hcmv or ccmv or human herpesvirus 5 or human herpes virus 5 or human herpesvirus type 5 or human herpes virus type 5 or human betaherpesvirus 5 or human beta-herpesvirus 5 or HHV 5 or HHV5 or salivary gland virus$ or cytomegalic).ab.	6201
8	exp Cytomegalovirus Infections/ and (Cytomegalovir$ or cmv or hcmv or ccmv or human herpesvirus 5 or human herpes virus 5 or human herpesvirus type 5 or human herpes virus type 5 or human betaherpesvirus 5 or human beta-herpesvirus 5 or HHV 5 or HHV5 or salivary gland virus$ or cytomegalic).ab.	22497
9	Cytomegalovirus antibody/ and (Cytomegalovir$ or cmv or hcmv or ccmv or human herpesvirus 5 or human herpes virus 5 or human herpesvirus type 5 or human herpes virus type 5 or human betaherpesvirus 5 or human beta-herpesvirus 5 or HHV 5 or HHV5 or salivary gland virus$ or cytomegalic).ab.	1500

10	congenital cytomegalovirus infection/ and (Cytomegalovir$ or cmv or hcmv or ccmv or human herpesvirus 5 or human herpes virus 5 or human herpesvirus type 5 or human herpes virus type 5 or human betaherpesvirus 5 or human beta-herpesvirus 5 or HHV 5 or HHV5 or salivary gland virus$ or cytomegalic).ab.	77
11	(Cytomegalovir$ or cmv or hcmv or ccmv or human herpesvirus 5 or human herpes virus 5 or human herpesvirus type 5 or human herpes virus type 5 or human betaherpesvirus 5 or human beta-herpesvirus 5 or HHV 5 or HHV5 or salivary gland virus$).ti.	32943
12	Cytomegalovir$.ab. /freq=2	5840
13	Cytomegalovir$.af. and (cmv or hcmv or ccmv or hhv 5 or hhv5).ab. /freq=2	28410
14	(Cytomegalovir$ or cmv or hcmv or ccmv or human herpesvirus 5 or human herpes virus 5 or human herpesvirus type 5 or human herpes virus type 5 or human betaherpesvirus 5 or human beta-herpesvirus 5 or HHV 5 or HHV5 or salivary gland virus$).ab. and (article-in-press or conference abstract or in-process).st.	19525
15	or/1-14	64306
16	Cytomegalovirus infection/ep	1263
17	congenital cytomegalovirus infection/ep	13
18	Seroepidemiology/	4292
19	Congenital infection/ep	195
20	Congenital disorder/ep	577
21	Intrauterine infection/ep	168
22	Seroprevalence/	22659
23	Prevalence/ or Incidence/	1121561

24	mortality/ or childhood mortality/ or embryo mortality/ or fetus mortality/ or hospital mortality/ or infant mortality/ or mortality rate/ or perinatal mortality/ or premature mortality/ or prenatal mortality/ or standardized mortality ratio/	899892
25	Cytomegalovirus seropositivity/	1
26	Infection rate/	31994
27	Newborn Screening/ or Prenatal Screening/	27988
28	dried blood spot testing/	4132
29	Virus Shedding/	7138
30	(prevalence$ or prevalent or incidence$ or incident or mortalit$ or epidemiolog$ or morbidity or seroepidemiolog$ or seroprevalen$ or seroinciden$ or serosurvey$ or sero survey or serosurveillance or sero surveillance).ti,ab,kw.	3610756
31	(cases adj4 (birth$ or year$ or positive or symptomatic or asymptomatic)).ti,ab.	149280
32	(CMV cases or HCMV cases or CCMV cases).ti,ab.	86
33	((transmission or diagnosis) adj4 rate$).ti,ab.	24869
34	(transmission adj3 risk).mp.	16642
35	(infection rate$ or disease rate$).ti,ab,kw.	36351
36	Frequency.ti,ab.	1090458
37	((neonat$ or newborn$ or prenatal or maternal) adj2 screening).ti,ab.	23777
38	Shedding.ti,ab,kw.	31222
39	or/16-38	5082459
40	cohort analysis/	644864
41	exp longitudinal study/	148767
42	observational study/	215825

43	retrospective study/	1001216
44	exp "medical record review"/	132556
45	prospective study/	646852
46	cross-sectional study/	383354
47	exp case control study/	183174
48	Factual database/	24898
49	Major clinical study/	3956032
50	Controlled study/	7834149
51	(Long-term or observational or cohort? or longitudinal or prospective or retrospective or comparative or case-control or cross-sectional or followup or survey$).ti.	1215504
52	(real-world or real life).ti.	31742
53	(cohort analys$ or cohort stud$ or longitudinal stud$ or chart review$ or medical record review$ or observational stud$ or retrospective analys$ or retrospective chart review? or retrospective clinical stud$ or retrospective cohort$ or retrospective stud$ or retrospective database$ or retrospective observational$ or cross-sectional stud$ or cross-sectional setting$ or case control stud$ or case control setting or nested case control stud$ or matched case-control stud$ or case series).kw.	49207
54	(cohort adj (study or studies or analy$)).ti,ab.	332858
55	(cohort$ adj2 (birth$ or newborn$)).ti,ab,kw.	24174
56	((longitudinal or cross sectional or case-control or pragmatic or large scale) adj (study or studies)).ti,ab.	477968
57	((observational or retrospective or prospective) adj3 (study or studies or cohort$1 or data$ or analys#s)).ti,ab.	1389623

58	((real world or real life) adj3 (data$ or setting or cohort$ or stud$ or experience$ or result$)).ti,ab.	46654
59	(data adj (report$ or data system$1)).ti,ab.	16238
60	Electronic Medical Record/	57789
61	(chart review or medical record review or (review adj5 patient records) or case series or consecutive patients or nonconsecutive patients).ti,ab,kw.	445053
62	((Population-based or nationwide or community based or national or hospital based) adj3 (stud$3 or cohort$ or data$ or register$ or database$ or sample$)).ti,ab.	324732
63	(Database analys#s or database stud$ or claims data$).ti,ab.	34877
64	(single center or single centre or single institution or hospital$).ti.	457582
65	or/40-64	12016316
66	Disease registry/	15355
67	register/	114821
68	(registries or registry or disease regist$3 or register$1 or national regist$).ti,ab,kw.	316380
69	or/66-68	335238
70	exp Health survey/	227776
71	exp disease surveillance/	30986
72	Population research/	110899
73	Newborn screening/	19532
74	dried blood spot testing/	4132
75	(health survey$1 or survey$1 or surveillance).ti,ab,hw.	1708256
76	Mass screening/ or Screening/	234734
77	Newborn screening/	19532

78	Prenatal screening/	8700
79	((neonat$ or newborn$ or prenatal or maternal) adj3 screen$).ti,ab.	27260
80	((CMV or HCMV or CCMV or cytomegalovirus) adj3 screen$).ti,ab.	923
81	Seroepidemiology/	4292
82	or/70-81	2071381
83	65 or 69 or 82	13301913
84	(exp Animal/ or animal experiment/ or nonhuman/) not (exp Human/ or Human experiment/)	6611415
85	animal experiment/ and (mouse or mice or murine or rat or rats or rabbit or rabbits or cat or cats or dog or dogs or swine or porcine or pig or pigs or piglet or piglets or lamb or lambs or cattle or bovine or monkey or monkeys).ti.	1093694
86	or/84-85	6663980
87	83 not 86	10542195
88	exp Mathematical model/	413479
89	Simulation/	186072
90	model$.kw.	285315
91	(model or models or modelling or modeling).ti. not (((mouse or mice or murine or rat or rats or rabbit or rabbits or cat or cats or dog or dogs or swine or porcine or pig or pigs or piglet or piglets or lamb or lambs or cattle or bovine or monkey or monkeys or rhesus or macaque$ or nonhuman or "in vitro" or animal$ or organism$) adj3 model$).ti,kw. or exp Animal Model/)	468758
92	((mathematic$ or statistic$ or transmission$ or epidemiolog$ or epidemic$ or vaccination$ or immuni$ or theoretical$) adj3 model$).ti,ab,kw.	152547
93	((linear or nonlinear or static$ or dynamic$ or explicit or implicit or discrete$ or continuous$ or deterministic$ or probabilistic$ or stochastic$ or deductive or inductive or floating or strateg$ or	261987

	nonstrateg$ or conceptual$ or quantitative$ or qualitative$ or individual$ or structured or catalytic) adj3 model$).ti,ab,kw.	
94	or/88-93	1331021
95	94 not ((exp Animal/ or animal experiment/ or nonhuman/) not (exp Human/ or Human experiment/))	1104765
96	95 not (animal experiment/ and (mouse or mice or murine or rat or rats or rabbit or rabbits or cat or cats or dog or dogs or swine or porcine or pig or pigs or piglet or piglets or lamb or lambs or cattle or bovine or monkey or monkeys).ti.)	1101397
97	87 or 96	11175572
98	Systematic Review/	273959
99	exp Meta Analysis/	203612
100	biomedical technology assessment/	14745
101	((meta adj analy$) or metaanalys$).tw.	246352
102	(systematic adj2 (review$1 or overview$1)).tw.	246106
103	(cochrane or embase or psychlit or psyclit or psychinfo or psycinfo or cinahl or cinhal or science citation index or bids or cancerlit or biosis or lilacs or web of science or scopus).ab.	219883
104	(reference lists or bibliograph$ or hand search$ or manual search$ or relevant journals).ab.	53874
105	(selection criteria or eligibility criteria or data extraction).ab. and review.pt.	34580
106	(((systematic$ or methodologic or quantitative or integrative or collaborative) adj5 (review or overview)) or technology assessment or bibliographic study).ti.	178339
107	or/98-106	567984
108	(letter or editorial).pt.	1832830
109	(exp Animal/ or animal experiment/ or nonhuman/) not (exp Human/ or Human experiment/)	6611415

110	animal experiment/ and (mouse or mice or murine or rat or rats or rabbit or rabbits or cat or cats or dog or dogs or swine or porcine or pig or pigs or piglet or piglets or lamb or lambs or cattle or bovine or monkey or monkeys).ti.	1093694
111	or/108-110	8430366
112	107 not 111	545967
113	exp Australia/ or Australian/	178801
114	Australia$.ti,ab,kw,jw.	306040
115	exp Japan/	174135
116	"japanese (people)"/ or "Japanese (people)"/	29902
117	Japan$.ti,ab,kw,jw.	668925
118	Israel/ or Israeli/	32304
119	Israel$.ti,ab,kw,jw.	49037
120	or/113-119	1101760
121	exp Canada/	186242
122	Canada.ti,ab,kw.	116235
123	Canadian$1.ti,ab,jw.	242240
124	(CADTH or pCODR or INESSS).ti,ab.	438
125	(british columbia or alberta$ or saskatchewan or manitoba$ or ontario or quebec or new brunswick or nouveau brunswick or nova scotia or nouvelle ecosse or prince edward island or newfoundland or labrador or nunavut or nwt or northwest territories or yukon or nunavik or inuvialuit).ti,ab,kw,jw.	97346
126	(Abbotsford or Airdrie or Ajax or Aurora or Barrie or Belleville or Blainville or Brampton or Brantford or Brossard or Burlington or Burnaby or Caledon or Calgary or Cape Breton or Chatham Kent or Chilliwack or Clarington or Coquitlam or Drummondville or Edmonton or Fredericton or	175870

	Fort McMurray or Gatineau or Granby or Grande Prairie or Sudbury or Guelph or Halton Hills or Iqaluit or Inuvik or Kamloops or Kawartha Lakes or Kelowna or Kingston or Kitchener or Langley or Laval or Lethbridge or Levis or Longueuil or Maple Ridge or Markham or Medicine Hat or Milton or Mirabel or Mississauga or Moncton or Montreal or Nanaimo or New Westminster or Newmarket or Niagara Falls or Norfolk County or North Bay or North Vancouver or North Vancouver or Oakville or Oshawa or Ottawa or Peterborough or Pickering or Port Coquitlam or Prince George or Quebec City or Red Deer or Regina or Repentigny or Richmond or Richmond Hill or Saanich or Saguenay or Saint John or Saint-Hyacinthe or Saint-Jean-sur-Richelieu or Saint-Jerome or Sarnia or Saskatoon or Sault Ste Marie or Sherbrooke or St Albert or St Catharines or St John's or Strathcona County or Surrey or Terrebonne or Thunder Bay or Toronto or Trois-Rivieres or Vancouver or Vaughan or ((Cambridge or (Halifax or Hamilton or London or Victoria or Waterloo or Welland or Whitby or Windsor)) not (UK or Britain or United Kingdom or England or Australia)) or Whitehorse or Winnipeg or Wood Buffalo or Yellowknife).ti,ab,kw.	
127	exp United States/	1239022
128	(united states or usa or us national or us census or us state$ or US cohort$ or US population or US adult$ or US children).ti,ab,kw.	553121
129	(US or (U adj S)).ti.	86245
130	(America$ not (Central America$ or South America$ or Latin America$)).ti.	126225
131	((midatlantic or mid-atlantic or middle-atlantic or Midwest$3 or northeast$3 or northwest$3 or southeast$3 or southern or southwest$3) adj (state$1 or region$ or us or usa)).ti,ab,kw.	19980
132	(Appalachia$ or great lakes or great plains or heartland or new England or pacific northwest or deep south or black belt or rust belt or district of Columbia or Washington DC or Alabama or Alaska or Arizona or Arkansas or little rock or California or san Francisco or San Diego or Los Angeles or Colorado or Connecticut or Florida or Hawaii or Honolulu or Idaho or Gainesville or Jacksonville or Tampa or Tallahassee or Georgia or Atlanta or Illinois or Chicago or Indiana or Indianapolis or West Lafayette or iowa or Kansas or Wichita or Kentucky or Louisiana or new Orleans or baton rouge or Shreveport or Maine or Orono or Johns Hopkins or Massachusetts or	690765

	Boston or Harvard or Michigan or Detroit or Ann Arbor or East Lansing or Minnesota or Minneapolis or Rochester or Montana or Missisippi or Missouri or Missoula or Nebraska or Nevada or Las Vegas or New Hampshire or New Jersey or New Mexico or New York or North Carolina or North Dakota or Ohio or Cincinnati or Oklahoma or Oregon or Illinois or Chicago or Maryland or Pennsylvania or Philadelphia or South Carolina or South Dakota or Tennessee or Nashville or Memphis or Texas or Houston or Utah or Vermont or Virginia or Rhode Island or Washington or Seattle or West Virginia or Wisconsin or Wyoming or Delaware or Mayo Clinic or AHRQ).ti,ab,kw.	
133	(((Birmingham or Montgomery) adj al) or Huntsville or anchorage or fairmbanks or Phoenix or Tuscon or Flagstaff or Berkeley or Stanford or Vail or Denver or Farmington or New Haven or Hartford or Wilmington or Newark or Miami or ((Athens or Augusta) adj ga) or Boise or Urbana or Evanston or Lexington or Louisville or Bardstown or (Scarborough adj me) or Bethesda or Baltimore or Rockville or (Worcester adj ma) or Burlington or St Paul or Saint Paul or (Jackson adj ms) or (Columbia adj mo) or Bozeman or Omaha or Lincoln or Columbus or Cleveland or Portland or Hershey or providence or Richmond or Washington).ti,ab,kw.	122899
134	Dollar$.ti,ab.	23472
135	(medicare or medicaid).ti,ab.	85900
136	North America/	35750
137	North America$.ti,ab,kw.	73994
138	exp North American/	168144
139	or/121-138	2780357
140	exp Europe/	1597527
141	exp European/ or exp EU Citizen/	181825
142	(Europe$3 or EU5).ti,ab,kw,jw.	1411562
143	exp France/ or Frenchman/	124269

144	France.ti,ab,kw.	95784
145	french.ti,ab,jw.	95290
146	exp Germany/ or "german (citizen)"/	188816
147	German$1.ti,ab,kw,jw.	250159
148	exp Italy/ or "italian (citizen)"/	119522
149	(Italy or Italian$).ti,ab,kw,jw.	235845
150	exp Spain/ or Spaniard/	100619
151	(Spain or Spanish or Spaniard$).ti,bt,ab,kw,jw.	140461
152	exp United Kingdom/ or exp British Citizen/	425149
153	(UK or U K).ti.	45801
154	(britain$ or (british$ not "british columbia") or united kingdom$ or (england$ not "new england") or northern ireland$ or northern irish$ or scotland$ or scottish$ or ((wales or "south wales") not "new south wales") or welsh$).ti,ab,kw,jw.	778592
155	(English adj3 (Society or Association or patients)).ti,ab.	3269
156	(English adj (population or hospital$1 or citizen$)).ti,ab.	937
157	"National Institute for Health and Care Excellence".ti,ab.	3497
158	(((Albania$ or Andorra$ or Armenia$ or Austria$ or Azerbaijan$ or Belarus$ or Belgium or Belgian$ or Bosnia$) and Herzegovin$) or Bulgaria$ or Croatia$ or Cyprus or Cyprian$ or Czechia$ or Denmark or Danish or Estonia$ or Finland or Finnish or Georgia$ or Greece or Greek$ or Hungary or Hungarian$ or Iceland$ or Ireland or Irish or Kosovo$ or Latvia$ or Liechtenstein or Lithuania$ or Luxembourg$ or Malta or Moldova$ or Monaco or Montenegro or Netherlands or Dutch$ or North Macedonia$ or Norway or Norwegian$ or Poland or Polish or Portugal or Portuguese or Romania$ or San Marino or Scandinavia$ or Serbia$ or Slovakia$ or Slovak	1167978

	Republic or Slovenia$ or Sweden or Swedish or Switzerland or Swiss or Turkey or Turkish or Ukrain$).ti,ab,kw,jw.	
159	(euro or euros or EUR).ti,ab.	26098
160	or/140-159	4485402
161	exp "South and Central America"/	241347
162	exp South American/ or exp Central American/	16973
163	(Latin? America$ or Latinoamerica$ or South America$ or Central America$ or Caribbean$).ti,ab,od,kw,jw.	102565
164	(Argentina or Argentinian$ or Bolivia$ or Brazil$ or Brasil$ or Chile or Chilean$ or Colombia$ or Ecuador or Ecuadorian$ or Paraguay$ or Peru or Peruvian$ or Urugay$ or Venezuela$ or Costa Rica$ or El Salvador or Salvadorian$ or Guatemala$ or Guatemaltec$ or Hondura$ or Mexico or Mexican$ or Nicaragua$ or Panama$5 or Dominican Republic or Dominican$).ti,ab,od,kw,jw.	498238
165	(Buenos Aires or La Paz or Sucre or Rio de Janeiro or Sao Paulo or Santiago or Valparaiso or Concepcion or Bogota or Quito or Asuncion or Lima or Montevideo or Caracas or San Salvador or Tegucigalpa or Managua or Santo Domingo).ti,ab,jw.	61799
166	or/161-165	586107
167	(Global$2 or worldwide or world wide).ti,ab,hw.	878268
168	International.ti.	98547
169	or/167-168	968039
170	120 or 139 or 160 or 166 or 169	9001213
171	97 and 170	3620536
172	112 or 171	4076674
173	15 and 39	21369
174	172 and 173	4354

175	limit 174 to yr="2000 -Current"	3856
176	175 and conference abstract.pt,st.	1810
177	limit 176 to yr="2000 - 2016"	1150
178	175 not 177	2706
179	limit 178 to English language	2587
180	((neonat$ or newborn$ or prenatal or antenatal or postnatal or maternal) adj3 screen$).ti,ab.	30089
181	(reactivat$ or re-activat$).ti,ab,kw.	60009
182	Force of infection$.ti,ab,kw.	627
183	(Pregnancy loss$ or miscarriage$ or abortion$ or f?etal death$).ti,ab,kw.	102095
184	((targeted or universal or routine) adj2 screen$).ti,ab.	27346
185	(sequelae or long term effects).ti,ab,kw.	119946
186	((emotional$ or psychologic$ or psychosocial$ or social or psychometric or neuropsycholog$ or neurodevelopment$ or development$ or neurocognit$ or cognit$ or mental or language or auditory or audiolog$ or hearing) adj3 (outcome$ or function$ or performance or skills)).ti,ab.	375977
187	(cognitive abilit$ or cognitive disabilit$ or cognitive delay$ or mental development or developmental disabilit$ or developmental abnormalit$ or audiologic?? outcome$ or hearing deficits or hearing impairment or hearing loss or hearing abilit$ or phonologic?? outcome$ or auditory perception or speech recognition or word recognition or speech discrimination or language production or language delay or speech perception or language perception or language development or (language adj2 processing) or speech Intelligibility or speech development or verbal ability or (child adj2 development) or emotional problems or peer problems or executive function$ or working memory or cognitive flexibility or flexible thinking or psychological development or mental health or school performance).ti,ab,kw.	431617

188	(Polymerase Chain Reaction/ or Real-Time Polymerase Chain Reaction/ or (Polymerase Chain Reaction or PCR or test$ or screen$).ti,ab,kw.) and (urin$ or saliva$ or oral swab$ or blood spot$).ti,ab.	202074
189	Alethia$.ti,ab,kw.	5
190	or/180-189	1236930
191	39 or 190	6007986
192	(Polymerase Chain Reaction/ or Real-Time Polymerase Chain Reaction/ or (Polymerase Chain Reaction or PCR or test$ or screen$).ti,ab,kw.) and (urin$ or saliva$ or oral swab$ or blood spot$).ti,ab.	202074
193	Alethia$.ti,ab,kw.	5
194	((neonat$ or newborn$ or prenatal or antenatal or postnatal or maternal) adj3 screen$).ti,ab.	30089
195	((targeted or universal or routine) adj2 screen$).ti,ab.	27346
196	or/192-195	251933
197	65 or 69 or 82 or 94 or 196	14150449
198	197 not 86	11248765
199	170 and 198	3641527
200	112 or 199	4097103
201	15 and 191	26763
202	200 and 201	5185
203	limit 202 to yr="2000 -Current"	4607
204	limit 203 to yr="2000 - 2016"	3097
205	204 and conference abstract.pt,st.	1453
206	203 not 205	3154

207	limit 206 to English language	3006

Supplemental Table 6. Widened search strategy for LILACS

	LILACS (Latin American and Caribbean Health Sciences Literature)<br><br>(Medline not included)
**Search Platform:**	VHL – Virtual Library<br><br>https://lilacs.bvsalud.org/en/
**Date of Search:**	December 14, 2020
**Date Range Searched:**	2000-2020

#	Search Terms	Hits
1	((mh:("Cytomegalovirus") OR mh:("Cytomegalovirus Infections") OR tw:("Cytomegalovirus") OR tw:("cmv") OR tw:("hcmv") OR tw:("ccmv") OR tw:("citomegalovirus") OR tw:("human herpesvirus 5") OR tw:("human herpes virus 5") OR tw:("human herpesvirus type 5") OR tw:("human herpes virus type 5") OR tw:("HHV 5") OR tw:("HHV5") OR tw:("salivary gland virus")) AND (mh:("Cytomegalovirus Infections/EP") OR mh:("Cytomegalovirus Infections/MO") OR mh:("Virus Diseases/EP") OR mh:("Virus Diseases/MO") OR mh:("Infant, Newborn, Diseases/EP") OR mh:("Infant, Newborn, Diseases/MO") OR mh:("Infant, Premature, Diseases/EP") OR mh:("Infant, Premature, Diseases/MO") OR mh:("Disease Transmission, Infectious/SN") OR mh:("Infectious Disease Transmission, Vertical/SN") OR mh:("Virus Shedding") OR mh:("Prevalence") OR mh:("Incidence") OR mh:("mortality") OR mh:("Seroepidemiologic Studies") OR mh:("Neonatal Screening") OR tw:(prevalenc*) OR tw:("prevalent") OR tw:(incidenc*) OR tw:("incident") OR tw:(mortalit*) OR tw:(epidemiolog*) OR tw:(seroepidemiolog*) OR tw:(seroprevalen*) OR tw:(seroinciden*) OR tw:(serosurvey*) OR tw:("sero-survey") OR tw:("serosurveillance") OR tw:("sero surveillance") OR tw:(seropositiv*) OR tw:("CMV cases") OR tw:("HCMV cases") OR tw:("CCMV cases") OR tw:(transmission rate*) OR tw:(infection rate*) OR tw:(disease rate*) OR tw:("frequency") OR tw:("neonatal screening") OR tw:("newborn screening") OR tw:("shedding") OR tw:("prenatal screening") OR tw:("maternal screening") OR tw:("dried blood spot testing") OR tw:("targeted screening") OR tw:("universal screening") OR tw:("routine screening") OR tw:(reactivat*) OR tw:("re-activation") OR tw:("force of infection") OR tw:(pregnancy loss*) OR tw:(abortion*) OR	1266

	tw:(miscarriage*) OR tw:(fetal death*) OR tw:(foetal death*) OR tw:("sequelae") OR tw:("long term effects") OR tw:(emotional outcome*) OR tw:(psychological outcome*) OR tw:(social outcome*) OR tw:(neuropsychological outcome*) OR tw:(neurodevelopmental outcome*) OR tw:(developmental outcome*) OR tw:(neurocognitive outcome*) OR tw:(cognitive outcome*) OR tw:(mental outcome*) OR tw:(language outcome*) OR tw:(auditory outcome*) OR tw:(audiological outcome*) OR tw:(hearing outcome*) OR tw:(emotional function*) OR tw:(psychological function*) OR tw:(social function*) OR tw:(neuropsychological function*) OR tw:(neurodevelopmental function*) OR tw:(developmental function*) OR tw:(neurocognitive function*) OR tw:(cognitive function*) OR tw:(mental function*) OR tw:(language function*) OR tw:(auditory function*) OR tw:(audiological function*) OR tw:(hearing function*) OR tw:(emotional performance) OR tw:(psychological performance) OR tw:(social performance) OR tw:(neuropsychological performance) OR tw:(neurodevelopmental performance) OR tw:(developmental performance) OR tw:(neurocognitive performance) OR tw:(cognitive performance) OR tw:(mental performance) OR tw:(language performance) OR tw:(auditory performance) OR tw:(audiological performance) OR tw:(hearing performance) OR tw:(emotional skills) OR tw:(psychological skills) OR tw:(social skills) OR tw:(neuropsychological skills) OR tw:(neurodevelopmental skills) OR tw:(developmental skills) OR tw:(neurocognitive skills) OR tw:(cognitive skills) OR tw:(mental skills) OR tw:(language skills) OR tw:(auditory skills) OR tw:(audiological skills) OR tw:(hearing skills) OR tw:(cognitive abilit*) OR tw:(cognitive disabilit*) OR tw:(cognitive delay*) OR tw:("mental development") OR tw:(developmental disabilit*) OR tw:(developmental abnormalit*) OR tw:("hearing deficits") OR tw:("hearing impairment") OR tw:("hearing loss") OR tw:(hearing abilit*) OR tw:(phonological outcome*) OR tw:("auditory perception") OR tw:("speech recognition") OR tw:("word recognition") OR tw:("speech discrimination") OR tw:("language production") OR tw:(language delay*) OR tw:("speech perception") OR tw:("language perception") OR tw("language development") OR tw:("language processing") OR ("speech Intelligibility") OR tw:("speech development") OR tw:(verbal abilit*) OR tw:("child development") OR tw:("emotional problems") OR ("peer problems") OR tw:(executive function*) OR tw:("working memory") OR tw:(" cognitive flexibility") OR tw:("flexible thinking") OR ("psychological development") OR tw:("mental health") OR tw:("school performance") OR ((mh:("polymerase chain reaction") OR tw:("polymerase chain reaction") OR tw:("PCR") OR	

	tw:(test*) OR tw:(screen*)) AND (tw:(urin*) OR tw:(saliva*) OR tw:(oral swab*) OR tw:(blood spot*))) OR tw:(alethia*))) AND (db:("LILACS" OR "IBECS" OR "BINACIS" OR "CUMED" OR "SES-SP" OR "LIPECS" OR "BBO" OR "MedCarib" OR "BRISA" OR "colecionaSUS" OR "BDENF" OR "ARGMSAL"))	
2	((mh:("Cytomegalovirus") OR mh:("Cytomegalovirus Infections") OR tw:("Cytomegalovirus") OR tw:("cmv") OR tw:("hcmv") OR tw:("ccmv") OR tw:("citomegalovirus") OR tw:("human herpesvirus 5") OR tw:("human herpes virus 5") OR tw:("human herpesvirus type 5") OR tw:("human herpes virus type 5") OR tw:("HHV 5") OR tw:("HHV5") OR tw:("salivary gland virus")) AND (mh:("Cytomegalovirus Infections/EP") OR mh:("Cytomegalovirus Infections/MO") OR mh:("Virus Diseases/EP") OR mh:("Virus Diseases/MO") OR mh:("Infant, Newborn, Diseases/EP") OR mh:("Infant, Newborn, Diseases/MO") OR mh:("Infant, Premature, Diseases/EP") OR mh:("Infant, Premature, Diseases/MO") OR mh:("Disease Transmission, Infectious/SN") OR mh:("Infectious Disease Transmission, Vertical/SN") OR mh:("Virus Shedding") OR mh:("Prevalence") OR mh:("Incidence") OR mh:("mortality") OR mh:("Seroepidemiologic Studies") OR mh:("Neonatal Screening") OR tw:(prevalenc*) OR tw:("prevalent") OR tw:(incidenc*) OR tw:("incident") OR tw:(mortalit*) OR tw:(epidemiolog*) OR tw:(seroepidemiolog*) OR tw:(seroprevalen*) OR tw:(seroinciden*) OR tw:(serosurvey*) OR tw:("sero-survey") OR tw:("serosurveillance") OR tw:("sero surveillance") OR tw:(seropositiv*) OR tw:("CMV cases") OR tw:("HCMV cases") OR tw:("CCMV cases") OR tw:(transmission rate*) OR tw:(infection rate*) OR tw:(disease rate*) OR tw:("frequency") OR tw:("neonatal screening") OR tw:("newborn screening") OR tw:("shedding") OR tw:("prenatal screening") OR tw:("maternal screening") OR tw:("dried blood spot testing") OR tw:("targeted screening") OR tw:("universal screening") OR tw:("routine screening") OR tw:(reactivat*) OR tw:("re-activation") OR tw:("force of infection") OR tw:(pregnancy loss*) OR tw:(abortion*) OR tw:(miscarriage*) OR tw:(fetal death*) OR tw:(foetal death*) OR tw:("sequelae") OR tw:("long term effects") OR tw:(emotional outcome*) OR tw:(psychological outcome*) OR tw:(social outcome*) OR tw:(neuropsychological outcome*) OR tw:(neurodevelopmental outcome*) OR tw:(developmental outcome*) OR tw:(neurocognitive outcome*) OR tw:(cognitive outcome*) OR tw:(mental outcome*) OR tw:(language outcome*) OR tw:(auditory outcome*) OR tw:(audiological outcome*) OR tw:(hearing outcome*) OR tw:(emotional function*) OR tw:(psychological function*) OR tw:(social function*) OR tw:(neuropsychological function*) OR tw:(neurodevelopmental function*) OR	882

	tw:(developmental function*) OR tw:(neurocognitive function*) OR tw:(cognitive function*) OR tw:(mental function*) OR tw:(language function*) OR tw:(auditory function*) OR tw:(audiological function*) OR tw:(hearing function*) OR tw:(emotional performance) OR tw:(psychological performance) OR tw:(social performance) OR tw:(neuropsychological performance) OR tw:(neurodevelopmental performance) OR tw:(developmental performance) OR tw:(neurocognitive performance) OR tw:(cognitive performance) OR tw:(mental performance) OR tw:(language performance) OR tw:(auditory performance) OR tw:(audiological performance) OR tw:(hearing performance) OR tw:(emotional skills) OR tw:(psychological skills) OR tw:(social skills) OR tw:(neuropsychological skills) OR tw:(neurodevelopmental skills) OR tw:(developmental skills) OR tw:(neurocognitive skills) OR tw:(cognitive skills) OR tw:(mental skills) OR tw:(language skills) OR tw:(auditory skills) OR tw:(audiological skills) OR tw:(hearing skills) OR tw:(cognitive abilit*) OR tw:(cognitive disabilit*) OR tw:(cognitive delay*) OR tw:("mental development") OR tw:(developmental disabilit*) OR tw:(developmental abnormalit*) OR tw:("hearing deficits") OR tw:("hearing impairment") OR tw:("hearing loss") OR tw:(hearing abilit*) OR tw:(phonological outcome*) OR tw:("auditory perception") OR tw:("speech recognition") OR tw:("word recognition") OR tw:("speech discrimination") OR tw:("language production") OR tw:(language delay*) OR tw:("speech perception") OR tw:("language perception") OR tw("language development") OR tw:("language processing") OR ("speech Intelligibility") OR tw:("speech development") OR tw:(verbal abilit*) OR tw:("child development") OR tw:("emotional problems") OR ("peer problems") OR tw:(executive function*) OR tw:("working memory") OR tw:(" cognitive flexibility") OR tw:("flexible thinking") OR ("psychological development") OR tw:("mental health") OR tw:("school performance") OR ((mh:("polymerase chain reaction") OR tw:("polymerase chain reaction") OR tw:("PCR") OR tw:(test*) OR tw:(screen*)) AND (tw:(urin*) OR tw:(saliva*) OR tw:(oral swab*) OR tw:(blood spot*))) OR tw:(alethia*))) AND (db:("LILACS" OR "IBECS" OR "BINACIS" OR "CUMED" OR "BBO" OR "LIPECS" OR "SES-SP" OR "BRISA" OR "colecionaSUS" OR "ARGMSAL")) AND (year_cluster:[2000 TO 2020])	
3	((mh:("Cytomegalovirus") OR mh:("Cytomegalovirus Infections") OR tw:("Cytomegalovirus") OR tw:("cmv") OR tw:("hcmv") OR tw:("ccmv") OR tw:("citomegalovirus") OR tw:("human herpesvirus 5") OR tw:("human herpes virus 5") OR tw:("human herpesvirus type 5") OR tw:("human herpes virus type 5") OR tw:("HHV 5") OR tw:("HHV5") OR tw:("salivary gland virus")) AND (mh:("Cytomegalovirus	186

	Infections/EP") OR mh:("Cytomegalovirus Infections/MO") OR mh:("Virus Diseases/EP") OR mh:("Virus Diseases/MO") OR mh:("Infant, Newborn, Diseases/EP") OR mh:("Infant, Newborn, Diseases/MO") OR mh:("Infant, Premature, Diseases/EP") OR mh:("Infant, Premature, Diseases/MO") OR mh:("Disease Transmission, Infectious/SN") OR mh:("Infectious Disease Transmission, Vertical/SN") OR mh:("Virus Shedding") OR mh:("Prevalence") OR mh:("Incidence") OR mh:("mortality") OR mh:("Seroepidemiologic Studies") OR mh:("Neonatal Screening") OR tw:(prevalenc*) OR tw:("prevalent") OR tw:(incidenc*) OR tw:("incident") OR tw:(mortalit*) OR tw:(epidemiolog*) OR tw:(seroepidemiolog*) OR tw:(seroprevalen*) OR tw:(seroinciden*) OR tw:(serosurvey*) OR tw:("sero-survey") OR tw:("serosurveillance") OR tw:("sero surveillance") OR tw:(seropositiv*) OR tw:("CMV cases") OR tw:("HCMV cases") OR tw:("CCMV cases") OR tw:(transmission rate*) OR tw:(infection rate*) OR tw:(disease rate*) OR tw:("frequency") OR tw:("neonatal screening") OR tw:("newborn screening") OR tw:("shedding") OR tw:("prenatal screening") OR tw:("maternal screening") OR tw:("dried blood spot testing") OR tw:("targeted screening") OR tw:("universal screening") OR tw:("routine screening") OR tw:(reactivat*) OR tw:("re-activation") OR tw:("force of infection") OR tw:(pregnancy loss*) OR tw:(abortion*) OR tw:(miscarriage*) OR tw:(fetal death*) OR tw:(foetal death*) OR tw:("sequelae") OR tw:("long term effects") OR tw:(emotional outcome*) OR tw:(psychological outcome*) OR tw:(social outcome*) OR tw:(neuropsychological outcome*) OR tw:(neurodevelopmental outcome*) OR tw:(developmental outcome*) OR tw:(neurocognitive outcome*) OR tw:(cognitive outcome*) OR tw:(mental outcome*) OR tw:(language outcome*) OR tw:(auditory outcome*) OR tw:(audiological outcome*) OR tw:(hearing outcome*) OR tw:(emotional function*) OR tw:(psychological function*) OR tw:(social function*) OR tw:(neuropsychological function*) OR tw:(neurodevelopmental function*) OR tw:(developmental function*) OR tw:(neurocognitive function*) OR tw:(cognitive function*) OR tw:(mental function*) OR tw:(language function*) OR tw:(auditory function*) OR tw:(audiological function*) OR tw:(hearing function*) OR tw:(emotional performance) OR tw:(psychological performance) OR tw:(social performance) OR tw:(neuropsychological performance) OR tw:(neurodevelopmental performance) OR tw:(developmental performance) OR tw:(neurocognitive performance) OR tw:(cognitive performance) OR tw:(mental performance) OR tw:(language performance) OR tw:(auditory performance) OR tw:(audiological performance) OR tw:(hearing	

	performance) OR tw:(emotional skills) OR tw:(psychological skills) OR tw:(social skills) OR tw:(neuropsychological skills) OR tw:(neurodevelopmental skills) OR tw:(developmental skills) OR tw:(neurocognitive skills) OR tw:(cognitive skills) OR tw:(mental skills) OR tw:(language skills) OR tw:(auditory skills) OR tw:(audiological skills) OR tw:(hearing skills) OR tw:(cognitive abilit*) OR tw:(cognitive disabilit*) OR tw:(cognitive delay*) OR tw:("mental development") OR tw:(developmental disabilit*) OR tw:(developmental abnormalit*) OR tw:("hearing deficits") OR tw:("hearing impairment") OR tw:("hearing loss") OR tw:(hearing abilit*) OR tw:(phonological outcome*) OR tw:("auditory perception") OR tw:("speech recognition") OR tw:("word recognition") OR tw:("speech discrimination") OR tw:("language production") OR tw:(language delay*) OR tw:("speech perception") OR tw:("language perception") OR tw("language development") OR tw:("language processing") OR ("speech Intelligibility") OR tw:("speech development") OR tw:(verbal abilit*) OR tw:("child development") OR tw:("emotional problems") OR ("peer problems") OR tw:(executive function*) OR tw:("working memory") OR tw:(" cognitive flexibility") OR tw:("flexible thinking") OR ("psychological development") OR tw:("mental health") OR tw:("school performance") OR ((mh:("polymerase chain reaction") OR tw:("polymerase chain reaction") OR tw:("PCR") OR tw:(test*) OR tw:(screen*)) AND (tw:(urin*) OR tw:(saliva*) OR tw:(oral swab*) OR tw:(blood spot*))) OR tw:(alethia*))) AND (db:("LILACS" OR "IBECS" OR "BINACIS" OR "CUMED" OR "BBO" OR "LIPECS" OR "SES-SP" OR "BRISA" OR "colecionaSUS" OR "ARGMSAL") AND la:("en")) AND (year_cluster:[2000 TO 2020])	

2. Supplement 2. Inclusion and exclusion criteria

Supplemental Table 7. PICO(+) framework for developing the search strategy and initial eligibility criteria; stages: abstract review, full text review, up to extraction

Category	Inclusion criteria	Exclusion criteria
Population	General population (latent CMV stage), all ages	Blood and solid organ donors, transplant recipients

	Toddlers/children 0-5 years of age (female and male)<br><br>Adolescents and females/males 9-40 years of age<br><br>HIV-positive mothers and HIV-infected babies<br><br>Other specific subpopulations or immunocompromised groups	High risk, immunosuppressed groups
Intervention	No restriction	No additional criteria specified
Comparator	No restriction	No additional criteria specified
Outcomes	<u>Epidemiology/seroepidemiology of cytomegalovirus (both CMV and cCMV)/infections</u><br><br>▪ Seroprevalence, prevalence<br>▪ Incidence, force of infection<br>▪ Mortality<br>▪ Infection rate<br>▪ Prevalence of CMV shedding (includes demographic breakdown)<br>▪ Vertical (pregnant women to fetus/child, child to adults) and horizontal (among adults) transmission rate<br>▪ Morbidity<br>▪ Long-term sequelae, long-term effects<br>▪ Pregnancy loss<br>▪ (including demographic breakdown: age distribution, serostatus, immunocompromised status, social status, education level, race/ethnicity	No additional criteria specified
Study types	Seroepidemiologic studies, epidemiologic studies<br><br>Cross-sectional, longitudinal, surveillance studies, registries<br><br>Mathematical models<br><br>Systematic reviews, meta-analyses	Clinical trials<br><br>Form of publication: comments, letters, editorials (full text review)
Language	English	Other than English
Country	Australia<br><br>Latin America, particularly Brazil<br><br>Canada<br><br>Europe (includes entirety of Europe and was not limited to EU5 [France, Germany, Italy, Spain, United Kingdom])<br><br>Israel<br><br>Japan<br><br>United States<br><br>Global (international, worldwide)	No additional criteria specified

Search time frame	- 2000-2020 (journal articles)<br>- 2017-2020 (conference abstracts)	No additional criteria specified
Data period to which the results relate	- 2010-2020 (journal articles)<br>- A period including any years from the 2000-2020 period (SLRs)	No additional criteria specified

27

Supplemental Table 8. PICO(+) framework for eligibility criteria; stages: Final inclusion to the SLR for the purpose of this report

Category	Inclusion criteria	Exclusion criteria
Population	Seroprevalence, prevalence: adults, women of reproductive age<br>Shedding, transmission: newborn, infants, children, adolescent, adults	-
Outcomes	▪ Seroprevalence, prevalence based on a non-IgM or IgG diagnostic method (eg, reverse transcription-polymerase chain reaction)<br>▪ Prevalence of CMV shedding (including demographic breakdown)<br>▪ Vertical (pregnant women to fetus/child, child to adults) and horizontal (among adults) transmission rate	▪ Incidence, force of infection<br>▪ Mortality<br>▪ Infection rate<br>▪ Long-term sequelae, long-term effects<br>▪ Pregnancy loss
Study/publication types	-	▪ Systematic reviews, meta-analyses<br>▪ Case series<br>▪ Conference abstracts/posters
Sample size	-	Sample size <100

3. **Supplement 3. Study characteristics**

Supplemental Table 9. Characteristics of the 29 studies included in the manuscript

Reference	Study design	Country	Study period	Study population	N	Age distribution	Sex	Diagnostic method
	**Europe**							
Arapović (2020) [32]	Observational	Bosnia and Herzegovina	2010-2019	Children and adults seeking care at the University Clinical Hospital Mostar (UCHM), pregnant women delivering at UCHM, and infants born between July 2011 and February 2013 at UCHM	Age 20-34: 1686; age 35-64: 1346; age >65: 385<br>Parity-no: 729; yes: 1180<br>Previous abortion- no: 1616; yes: 293<br>Pregnancy complications-Normal: 1384; Affected: 545<br>Gestational age: Term (> 37 w): 1831<br>Mode of delivery-Vaginal: 1665; C.S: 240<br>Birth weight (g)- < 3000: 240; 3000–4000:	≥1 year	Males, females	Serum samples were analyzed for CMV IgG antibodies using ELFA; cord blood specimens were tested using ELISA

					1419; > 4000: 244<br>Newborn complications-No: 1350; yes: 579			
Vilibić-Čavlek (2017) [48]	Prospective cohort	Croatia	2013-2015	Patients residing at all Croatian counties were tested at 2 large medical institutions (Croatian National Institute of Public Health and Clinical Hospital Center Zagreb)	Age 20-29: 431; age 30-29: 584; age 40-49: 478; age 50-59: 305; age 60+: 288<br>Hemodialysis patients: 314<br>Pregnant women with normal pregnancy: 238	Adults aged ≥20 years	Males, females	Consecutive serum samples were tested for presence of CMV specific IgG and IgM by ELISA and ELFA; positive samples were tested for IgG avidity by ELISA or ELFA
Antona (2017) [29]	Population-based seroprevalence survey, 2-stage stratified sampling design	France	2010	Individuals living in metropolitan France and attending private microbiological laboratories for blood testing	Male: 1230; female: 1306<br>Age 25-34: 751; age 35-49: 763	15-49 years	Males, females	CMV IgG ELISA

Grosjean (2014) [57]	Prospective	France	2014[a]	Children admitted to the emergency unit of Limoges Hospital in southern France and children who attended 6 daycare centers that greatly varied in size and characteristics	Children who attended six DCCs (day care centers): 256<br>Children admitted to the emergency unit (EU): 369	3 months-6 years	Males, females	RT-PCR targeting UL83 gene
Puccio (2014) [36]	Retrospective (chart review)	Italy	2012	Mothers of live babies born in the Department of Sciences for Health Promotion and Mother and Child Care of the University of Palermo	Overall: 797	16-51 years	Female s	Tested for anti-IgG and IgM antibodies during pregnancy
Barlinn (2014) [33]	Observational	Norway	2010-2011	Pregnant women from Drammen Hospital, Vestre Viken Hospital Trust Buskerud, and	General: 2000<br>Age 20-24: 281; age 25-29: 661; age 30-34: 637; age 35-39: 326	16-46 years	Female s	Serum samples were analyzed for anti-CMV IgG; anti-CMV IgM was assessed in samples from St. Olav's Hospital only

				St. Olav’s Hospital in Trondheim	General, Mid-Norway, IgM: 2000			
Plewik (2017) [35]	Observational	Poland	2015	Healthy women from the Biała Podlaska District	General: 175	16-35 years	Females	Blood samples were tested for presence of anti-CMV IgG by ELISA
Wujcicka (2014) [38]	Prospective	Poland	2010-2011	Pregnant women who attended the outpatient obstetric clinics and were treated at the Polish Mother’s Memorial Hospital Research Institute and the Ludwig Rydygier Hospital	Overall: 1250<br>Age 21-25: 199; age 26-30: 509; age 31-35: 362; age ≥ 36: 130<br>Education-higher: 667; secondary: 369; Primary and vocational: 144<br>Having children-No: 691; Yes: 479<br>Financial status-Average: 359; Good: 616; Unknown: 151<br>Risk of occupational contact with children-No: 799; Yes: 373	16-45 years	Females	Serum anti-HCMV IgG and IgM antibody levels were assayed by ELISA; IgG avidity was also tested

Siennicka (2017) [37]	Retrospective	Poland	2010-2011	Serum from women of reproductive age were selected from a serum bank maintained by the National Institute of Public Health-National Institute of Hygiene; samples were selected with the intent to represent the general population	Overall: 712<br>Age <30: 218; age 30–34: 105; age 35–39: 133; age 40–44: 114; age 45+: 142<br>Residence-Rural: 323; urban: 388<br>Region-Wielkopolskie: 177; Lubelskie: 122; Mazowieckie: 200; Świętokrzyskie: 116	Of childbearin g age	Female s	Serum samples were tested for CMV IgG antibodies
Gorun (2020) [34]	Retrospective	Romania	2015-2018	Pregnant women tested at SC Bioclinica srl, Timisoara, Romania	General: 7485<br>Urban: 3570; urban age 21-25: 526; urban age 26-30: 1331; urban age 31-35: 1057; urban age >35: 565<br>Rural: 1930; rural age <20: 185; rural age 21-25: 2509; rural age 26-30: 651; rural	NA	Female s	Serum samples were evaluated for anti-CMV IgG antibodies

					age 31-35: 391; rural > 35: 194			
Alari-Pahissa (2018) [46]	Prospective cross-sectional multicenter	Spain	2018[a]	Healthy adults from 3 hospitals integrated in the Spanish Network for MS Research (REEM)	General: 155	Adults (mean age: 37.6 years)	Males, females	Selected panel of immunological markers of differentiated/senescent T cells previously related to CMV infection were evaluated
Gonzalez-Quijada (2014) [54]	Case-control	Spain	2011-2012	Hospitalized patients	Control: 290<br>Cases (patients with prevalent or incident coronary heart, cerebrovascular or peripheral artery, disease): 164	≥65 years	Males, females	Blood samples were analyzed for CMV IgG antibody by ELISA
Firth (2016) [53]	Prospective	United Kingdom	2016[a]	Elderly adults who participated in the Scottish Mental Survey of 1947 when aged 11 years	General: 1054	70 years	Males, females	CMV was measured in plasma samples (collected at aged 70 years) by ELISA
Maple (2020) [47]	Retrospective	United Kingdom	2012	Adult controls	Controls: 124	Adults	Males, females	CMV IgG, EBNA-1 IgG, and EBV VCA IgG were detected and quantified by ELISA

Abdel Hamid (2011) [45]	Unclear[b]	United Kingdom	2011[a]	British neonates, children, adults	Age- Less than 2 weeks: 176; 2 weeks to 5 years: 451; 6 to 10 years: 211;25 years to 35 years: 125	<2 weeks-10 years; 25-35 years	NA	Serum samples: EIA for CMV IgG and IgM<br>Urine samples: rapid culture method
	**Latin America**							
Tiguman (2020) [52]	Cross-sectional	Brazil	2016	Adults that had previously participated in a major population-based survey carried out in the Manaus metropolitan region	Overall: 136<br>Nonwhite: 101<br>Health insurance-yes: 124<br>Malaria-yes: 132; Dengue-Yes: 129<br>Epstein-Barr vírus -No: 133	≥18 years	Males, females	Plasma samples were analyzed for anti-CMV IgG or IgM by ELISA
	**North America**							
Lamarre (2015) [43]	Prospective cohort	Canada	2010-2013	Pregnant women; serum samples were collected during the first, second, and third trimesters, as	Overall: 1938<br>Age 17-29: 763; age 30-34: 758; age 35-47: 412<br>Marital status-Married or common law:	17-47 years	Females	Serum samples were assessed for anti-CMV IgG antibodies by ELISA

				well as at delivery	1835; single or divorced: 101<br>Occupation- Nurse or midwife: 166; other or unknown: 1713<br>Education- Primary or secondary: 1911; post-secondary: 525; university: 1206<br>Household income (CAD)- \$0-59,999: 557; \$60,000-99,999: 705; ≥\$100,000: 586<br>Number of babies ever born- 0: 1075; ≥1: 863<br>Number of children still alive- 0: 1089; ≥1: 849<br>First language- French or English: 1435; other: 501			

					Country of birth- Canada or USA: 1315; other: 620			
Wizman (2016) [44]	Prospective	Canada	2012	Pregnant women who were delivering at Centre Hospitalier Universitaire Sainte-Justine in Montreal	Overall: 491<br>Age 18-30: 185; age 31-35: 186; age ≥36: 120<br>Born in Canada- Yes: 272; No: 219<br>Education level- Up to university: 188; university: 303<br>Family income- Low: 101; middle: 228; high: 150<br>Other children- 0: 229; 1: 171<br>Daycare Yes: 218; no: 273<br>Employment- None: 112; others: 301	>18 years	Female s	CMV serostatus was identified in first trimester blood samples using an automated enzyme immunoassay analyzer
Alvarado-Esquivel (2014) [39]	Cross-sectional	Mexico	2013	Pregnant women in a public primary healthcare center,	Overall: 343<br>Age 15-30: 198; age 31-43: 144	15-43 years	Female s	Cytomegalovirus IgM and IgG kit

				residing in Durango City	Birthplace Durango State: 319<br>Residence place: Durango State: 342<br>Residence area: urban: 325<br>Educational level- 7 to 12 years: 133; 13 or more years: 209<br>Unemployed: 110; employed: 233<br>Medium socio-economic level: 316<br>Month of pregnancy- 1 to 3: 117; 4 to 6: 153<br>Deliveries-Yes: 140; No: 202<br>Pregnancies: 3 or more: 228; 1 to 2: 115			

					Cesarean sections-none: 241; 1 to 2: 101<br>No miscarriages: 269			
Alvarado-Esquivel (2017) [40]	Case-control	Mexico	2011-2013	Pregnant women (24-42 weeks of pregnancy) suffering hypertensive disorders and proteinuria, attending the Department of Gynecology and Obstetrics of the General Hospital in Durango City, Mexico	Cases overall: 146; controls: 146	15-39 years	Females	Cytomegalovirus IgG kit
Alvarado-Esquivel (2018) [41]	Cross-sectional	Mexico	2014-2016	Pregnant women (1-9 months of pregnancy) were enrolled when attending their prenatal care consultations in 3 public health centers	Overall: 289<br>Age 20 or less: 126; age 21-30: 126<br>Ethnic group-Mestizo: 266<br>Birthplace: Aguascalientes State: 240	13-42 years	Females	ELFA, VIDAS CMV IgG and IgM

					Residence area-Urban: 206<br>Educational level-7- 12 years: 225<br>Occupation-Housewife: 230<br>High socio-economic level: 220			
De la Tejera-Hernández (2015) [51]	Case-control	Mexico	2013	Enrolled dentistry students	Overall: 176	Young adults (mean age: 21.5 years)	Males, females	Blood samples were assessed using a solid-phase, sequential chemiluminescent enzyme immunoassay
Dollard (2011) [42]	Observational (registry)	United States	2011	Women from the National Health and Nutrition Examination Survey III	Age 20-29: 1643; age 30-39: 1616; age 40-49: 1208	20-49 years	Females	CMV IgG was measured in sera using enzyme immunoassay and VIDAS automated test; CMV IgM was measured in sera using the VIDAS test
Stowell (2014) [56]	Cross-sectional	United States	2010[b]	Children	Overall: 161	0-47 months	Males, females	Serum was tested for anti-CMV IgG antibody; saliva and urine were tested for CMV DNA
Styles 2020 [30]	Cross-sectional	United States	2013	Adults in the Raleigh-Durham Chapel Hill metropolitan	General: 694<br>Age 18–29: 206; 30-39: 138; 40-49: 130; 50-59:	18-85 years	Males, females	Serum samples were tested for IgG antibody response to CMV by ELISA

				area in North Carolina	152; males: 264; females: 430			
Petersen (2020) [55]	Retrospective data analysis	United States	2011-2012; 2017-2018	Children in 2011-2012 and 2017-2018; data were collected from the continuous National Health and Nutrition Examination Surveys conducted by the National Center for Health Statistics	<u>2011-2012 cohort</u><br>Non-Hispanic White: 119; Non-Hispanic Black: 229; All Hispanic: 254<br>Less than High School diploma: 209; GED, HS diploma, associate degree, some college: 347; College degree or more: 124<br>Family income to poverty ratio- Below poverty level (<1,0): 296; At or above poverty level (≥1,0): 358<br><u>2017-2018 cohort</u><br>Non-Hispanic White: 233; Non-Hispanic Black: 136; All Hispanic: 133; Non-	1-5 years	Males, females	Serum samples were assessed for presence of CMV IgG antibodies

					Hispanic other/multiracial : 104<br>GED, HS diploma, associate degree, some college: 348; College degree or more: 130<br>Family income to poverty ratio- Below poverty level (<1,0): 188; At or above poverty level (≥1,0): 356			
	**Japan**							
Toriyabe (2017) [31]	Prospective cohort	Japan	2013-2015	Pregnant women (first trimester) with confirmed maternal primary infection	First trimester: 8469	16-45 years	Females	ELISA (IgM and IgG), RT-PCR of CMV DNA; performed on either a neonatal urine sample (< 1 week of life) or amniotic fluid sample
Takemoto (2016) [50]	Observational	Japan	2013	Neonates (born at ≥36 weeks of gestation)	Overall: 561	Neonates	Males, females	Umbilical cord blood was assessed for anti-CMV IgG antibodies by EIA

Takao (2020) [49]	Prospective cohort	Japan	2013-2018	Healthcare workers at the Osaka University Hospital	Overall: 1153; medical doctors: 386; nurses: 468; others: 299	Median age: 36 years	Males, females	Serum samples were tested for anti-CMV IgG

CMV, cytomegalovirus; EBNA-1, Epstein–Barr virus nuclear antigen 1; EBV, Epstein-Barr Virus; EIA, enzyme immunoassay; ELFA, enzyme-linked fluorescent assay; ELISA, enzyme-linked immunosorbent assay; IgG, immunoglobulin G; IgM, immunoglobulin M; NA, not available; RT-PCR, reverse transcription polymerase chain reaction.

[a]Publication date.

[b]Unclear information.
